# Self-Avoiding Gamma Peptide Nucleic Acids for Selective Targeting of RNA Secondary Structures

**DOI:** 10.1021/acschembio.5c00830

**Published:** 2026-03-17

**Authors:** Isha Dhami, Shivaji A. Thadke, J. Dinithi R. Perera, Ramesh Batwal, En Zheng, Savani W. Thrikawala, Babita Aryal, Danith H. Ly

**Affiliations:** Department of Chemistry and Institute for Biomolecular Design and Discovery (IBD), 6612 Carnegie Mellon University, 4400 Fifth Avenue, Pittsburgh, Pennsylvania 15213, United States

## Abstract

RNA molecules play essential roles in all aspects of cellular function, but their complex secondary and tertiary structures pose significant challenges for selective targeting. Traditional antisense strategies often avoid these structured regions, focused instead on unstructured sequences. In this study, we present an enhanced Self-Avoiding Molecular Recognition System designed to selectively recognize and bind structured RNA elements, offering an alternative approach for targeting biologically relevant RNA conformations with improved specificity and selectivity. This is achieved by incorporating established self-avoidance nucleobases (t and c) along with a deazapurine series (a and g)—designed to provide greater flexibility in fine-tuning binding affinity—into a conformationally preorganized gamma peptide nucleic acid backbone. Despite possessing self-complementary arms (*b* and *b’*), the system resists self-hybridization and selectively binds to the intended stem-loop RNA target (*bcb’*). Thermal stability measurements, electrophoretic mobility assays, and mismatch specificity analyses confirm the effectiveness of this approach, offering a general strategy for targeting structured RNA with precision.

## Graphical abstract

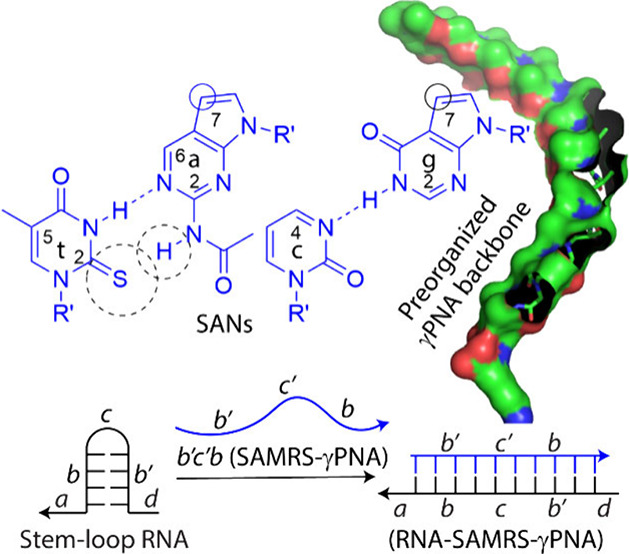

## Introduction

RNA molecules play crucial roles in cells beyond protein biosynthesis, including gene regulation, chromosome maintenance, and catalysis.^1^ Essential to these functions are their three-dimensional structures.^2^ Like proteins, RNAs adopt diverse secondary structures, such as stem-loops, bulges, and junctions, which subsequently self-assemble into complex molecular architectures.^3^ One such example is a pseudoknot (Figure 1), a compact, interwoven three-dimensional structure that plays key roles in gene regulation and is difficult to target using conventional small-molecule or antisense approaches. However, unlike proteins, RNA structures are more flexible and dynamic, often adopting multiple conformations,^2^ which makes rational design of selective small-molecule binders particularly challenging.

**Figure 1:**
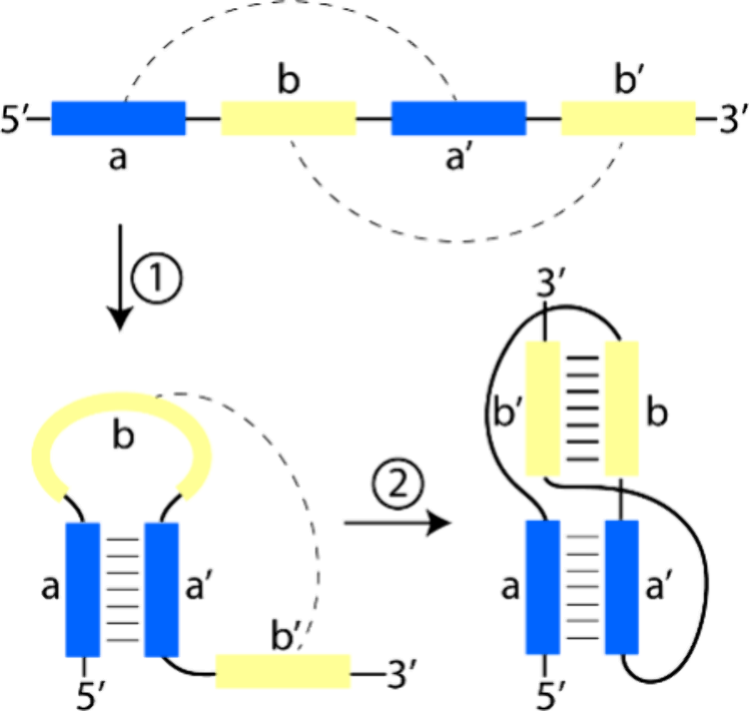
Formation of an RNA pseudoknot structure.

RNA targeting relies mainly on two strategies: small-molecule and antisense approaches, each with distinct appeals and setbacks. Small molecules offer therapeutic promise but are challenging to design, often requiring extensive screening and optimization.^4–6^ In contrast, oligonucleotides provide precise, sequence-specific recognition but struggle to bind structured RNAs due to kinetic and thermodynamic barriers.^7^ Peptide nucleic acids (PNAs), for example, have been used to target the pseudoknot in the SARS-CoV -1 ribosomal frameshifting site.^8^ Yet, this approach must balance the ability to unfold complex RNA structures with the need for high sequence specificity.

A more recent alternative leverages Hoogsteen base-pairing to recognize native RNA structures without requiring unfolding. Notably, the Rozner^9,10^ and Chen^11,12^ laboratories demonstrated this approach can modulate RNA not only in vitro but also in living cells. While promising for targeting continuous stem regions, its effectiveness against sequences with gaps or mismatches remains to be seen.

Among the most attractive yet challenging targets are stem-loops—prevalent secondary structures that frequently contribute to the assembly of larger, more complex tertiary motifs. Their high thermodynamic stability make them particularly difficult to disrupt during hybridization.^13^ One promising but less explored strategy is to target the entire length of the stem-loop regions of RNA. This can be accomplished by incorporating a specifically designed Self-Avoidance Molecular Recognition System (SAMRS)^14^ into a custom-engineered nucleic acid backbone.

Self-avoiding nucleobases (SANs), such as the 2-thiothymine (T^s^)/thiouracil (U^s^)-diaminopurine (D) pairs, were initially developed by Gamper^15,16^ for DNA/RNA systems and by Nielsen^17^ for use in peptide nucleic acids (PNAs) in the context of double-duplex invasion. Recently, Winssinger^18^ introduced the pseudo-C:G base-pair. Benner^14^ later extended this concept by modifying additional nucleobases, including the removal of the exocyclic amine groups from cytosine and guanine, as well as the carbonyl from thymine. Further advancements, such as the substitutions of cytosine and thymine with *N* ^4^-methylcytosine and 2-thiothymine, respectively, have proven effective in multiplex PCR applications. However, despite these elaborate chemical modifications, a SAMRS capable of effectively engaging RNA targets, especially those in their native (folded) states, has yet to be developed.

Here, we present an advanced SAMRS design based on the gamma peptide nucleic acids­(γPNA) backbone that is capable of binding both strands of the stem and the loop regions of RNA without forming secondary structure itself. When applied to RNA pseudoknot—a highly complex tertiary fold—this SAMRS-γPNA system enables sequence-specific and structure-selective RNA binding, addressing a challenge that has not been fully met by existing antisense approaches.

## Results

### Molecular Design

A major challenge in designing effective RNA-targeting oligonucleotides is preventing the formation of unintended secondary structures that can interfere with binding.^19^ To overcome this limitation, we reconfigured natural U-A and C-G hydrogen-bonding interactions into modified t-a and c-g pairs, where t denotes 2-thiothymine (Figure 2a and 2b). In the modified a, the C6-amine group of adenine was relocated to C2 and acetylated. This latter chemical modification had no impact on thermal stability or recognition specificity, while also simplifying solid-phase synthesis by eliminating the need for amine-group protection and deprotection steps. Following Benner’s approach,^14^ we removed the exocyclic amine groups from cytosine and guanine to create c and g, respectively, and further optimized RNA binding by introducing an N7→C7 substitution in g and a. This substitution was implemented in anticipation of the need for increased RNA binding free energy without increasing the risk of SAMRS-γPNA self-hybridization. Enhancement in RNA binding could be achieved by replacing c with a cytosine analog such as clamp-G,^20^ which forms five hydrogen-bonds with guanine in the RNA strand, but only three with g in the SAMRS-γPNA strand (Figure S1, Supporting Information). With respect to t, a, c, and g, each SAN maintains the ability to uniformly form two hydrogen-bonds with its complementary RNA bases (A, U, G, or C) (Figure 2c), thereby preserving both affinity and specificity during hybridization.

**Figure 2:**
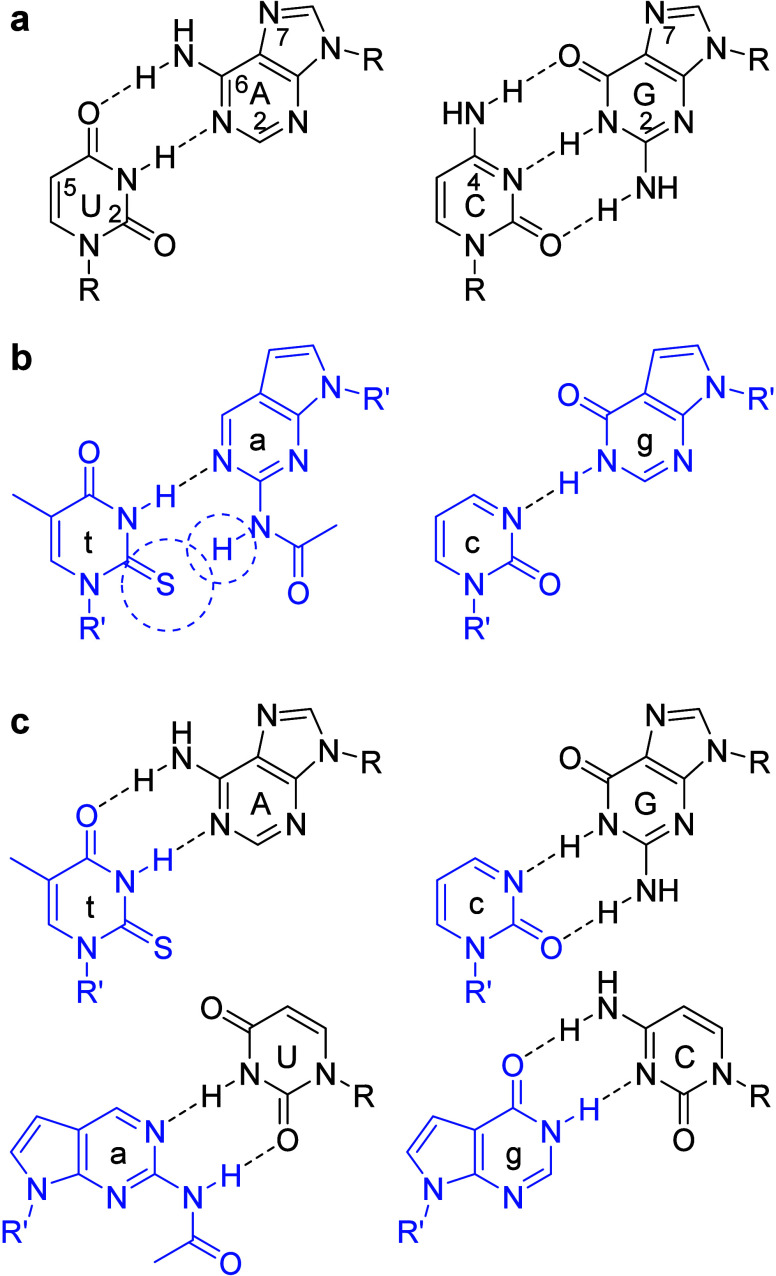
Hydrogen-bonding interactions of (a) natural nucleobases, (b) SANs (blue), and (c) SANs with RNA bases.

### Synthesis of SANs and the Corresponding SAMRS-γPNA Monomers

Eight chemical building blocks were prepared (Chart 1), consisting of four natural nucleobases (**1a**-**d**) and four SANs (**1e**-**h**). The natural nucleobases were synthesized using established protocols,^21^ while the synthesis of SANs is outlined in Scheme 1. Carboxymethylthiouracil (**3**) was obtained from 5-methyl-2-thiouracil (**2**) via a published procedure.^22^ The modified nucleobase c (**5**) was synthesized in two steps—alkylation followed by hydrolysis—from 2-hydroxypyrimidine (**4**). The guanine analog g (**7**) was synthesized from 7-deaza-chloropurine (**6**) in three steps involving alkylation, acetylation, and hydrogenation, the latter facilitating both dechlorination and benzyl ester reduction. Variant a (**9**) was synthesized similarly from compound **8**. Finally, the carboxymethyl nucleobases were coupled to an *L*-serine-derived Fmoc-γPNA backbone,^23,24^ followed by hydrolysis, yielding **1a**-**h** in reasonable yields (62–71%).

**Chart 1:**
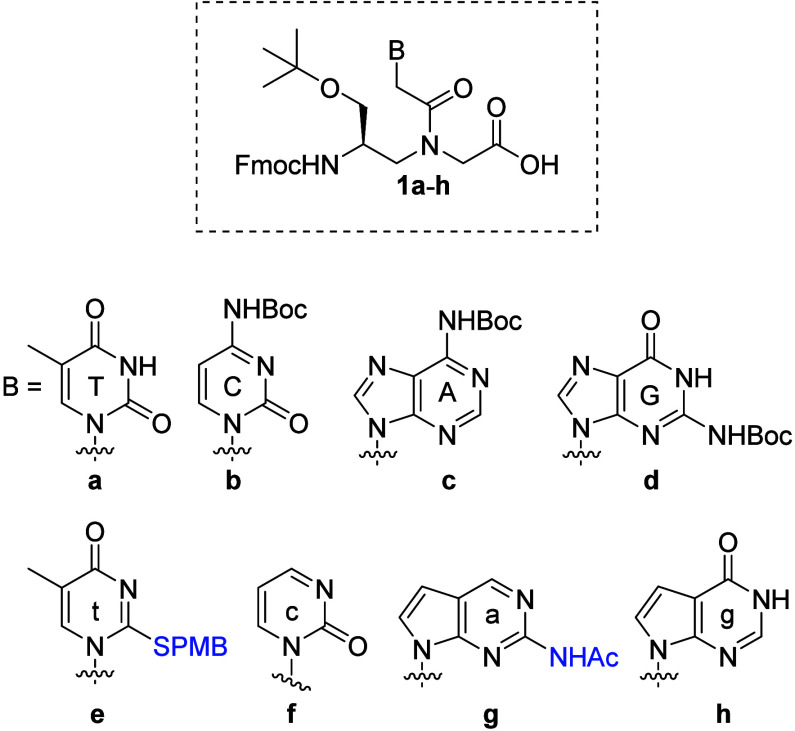


**Scheme 1:**
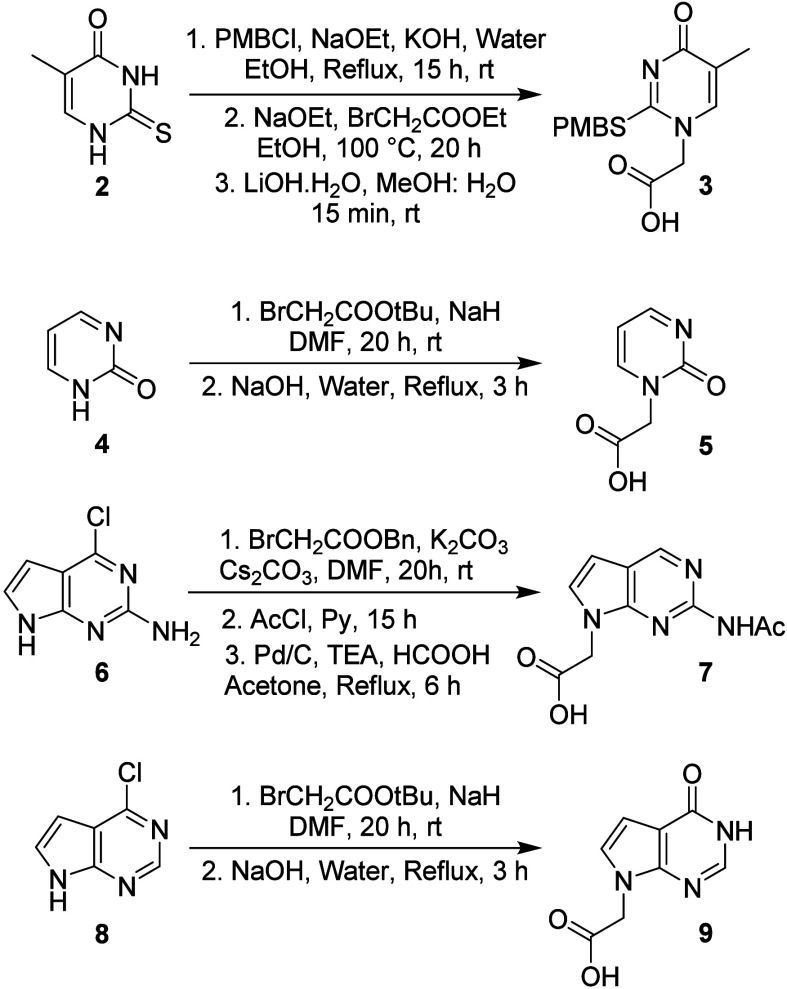


### Synthesis of γPNAs Containing Different Number of SAN Units

γPNA oligomers (P2 through P10 and P2’ through P10’), incorporating various SAN modifications—including sequences with four consecutive or alternating SANs—were synthesized on solid-support, along with achiral PNA controls (P1 and P1’) (Table 1)—without the γ-hydroxymethyl side chain. The oligomers were purified by reverse-phase HPLC and confirmed by MALDI-TOF (Figure S2 through **S21**). In the γPNA sequences, natural nucleobases are represented by uppercase letters (T, C, A, G), while SAN-modified units are denoted by lowercase letters and highlighted in blue for ease of visualization (t, c, a, g). Achiral PNA units are indicated by italicized uppercase letters (*T*, *C*, *A*, *G*).

**Table 1:**
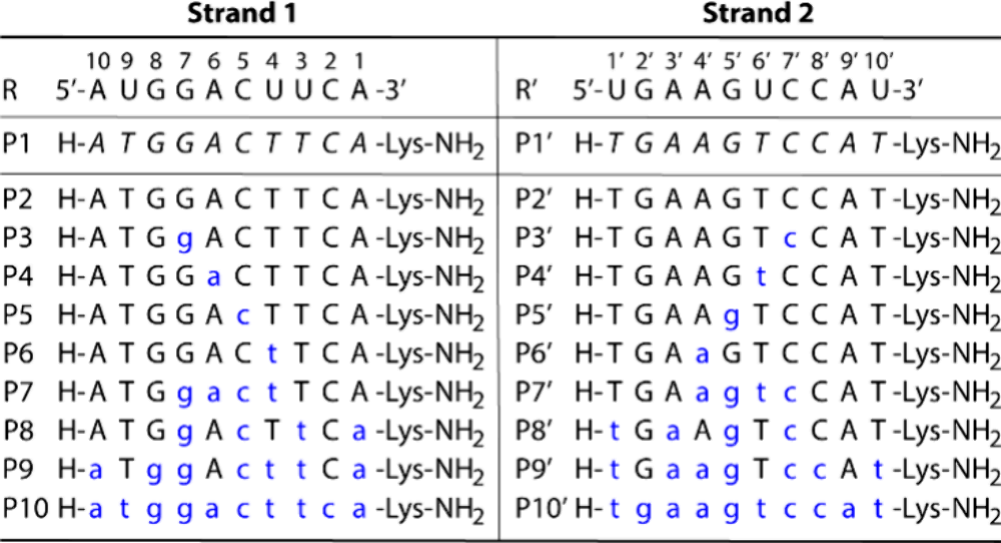
Sequence of RNA, PNA, and γPNA Oligomers^a^

a R and R’: RNA targets. P1 and P1’: classic (achiral) peptide nucleic acids containing natural nucleobases (italicized uppercase letters). P2 and P2’: serine-derived chiral γPNAs containing natural nucleobases (uppercase letters). P3 through P10 and P3′ through P10’: serine-derived chiral γPNAs containing a mixture of natural nucleobases (upper case letters) and SANs (lowercase letters and highlighted in blue).

### Effects of SANs on the Thermal Stability of γPNA-γPNA and γPNA-RNA Duplexes

To assess the impact of SANs on γPNA thermal stability, we conducted UV-melting experiments with complementary γPNA and RNA strands. Incorporation of individual SANs into γPNA-γPNA duplexes lowered the melting transitions (T_m_s) by ∼ 5.1/4.7 °C for a-t/t-a pairs and ∼14.4/16.0 °C for g-c/c-g pairs, compared to the natural counterparts (Table 2, Figure S22). In contrast, SANs had a milder destabilizing effect on γPNA-RNA duplexes (Table 3, Figure S23 and S24). Substituting A-U/U-A pairs with a-U/t-A reduced the T_m_s of γPNA-RNA duplexes by ∼1.8/1.8 °C, while substituting C-G/G-C with c-G/g-C led to a more substantial decrease of ∼8.7/9.3 °C. Nevertheless, this destabilization was still significantly less than that observed in γPNA-γPNA duplexes, aligning with expectations.

**Table 2:**
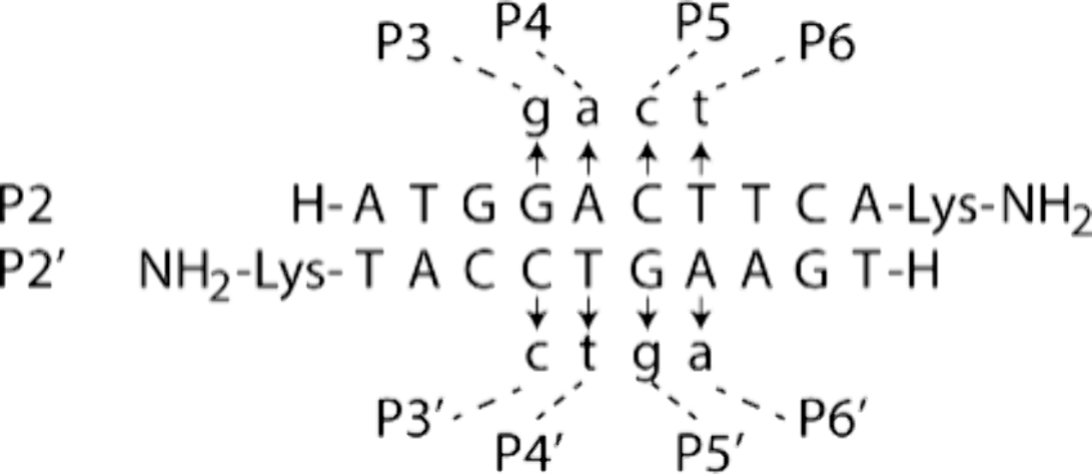
Effects of SAN Substitutions on the Thermal Stability of γPNA-γPNA Duplexes

Duplexes	Pairings	*T*_m_ (Δ*T*_m_), °C
P2–P2’	All natural nucleobases	88.5
P3–P3′	g<>c	74.1 (−14.4)
P4–P4’	a<>t	83.4 (−5.1)
P5–P5′	c<>g	72.5 (−16.0)
P6–P6’	t<>a	83.8 (−4.7)

**Table 3:**
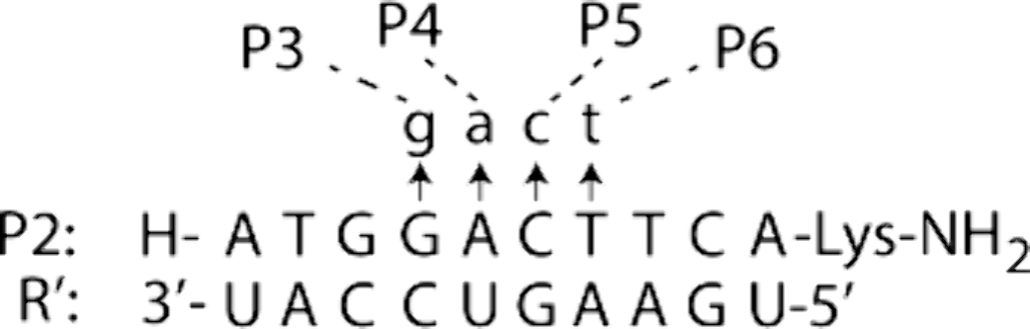
Effects of SAN Substitutions on the Thermal Stability of γPNA-RNA Duplexes

Duplexes	Pairings	T_m_ (DT_m_), °C
P2-R’	All natural nucleobases	72.9
P3-R’	g<>C	64.2 (−8.7)
P4-R’	a<>U	71.1 (−1.8)
P5-R’	c<>G	63.6 (−9.3)
P6-R’	t<>A	71.1 (−1.8)

The samples were prepared in 1× PBS buffer at a stoichiometric concentration of 2.5 μM per strand. The same conditions were applied to the measurements shown in Table 3 and Figure 3.

**Figure 3:**
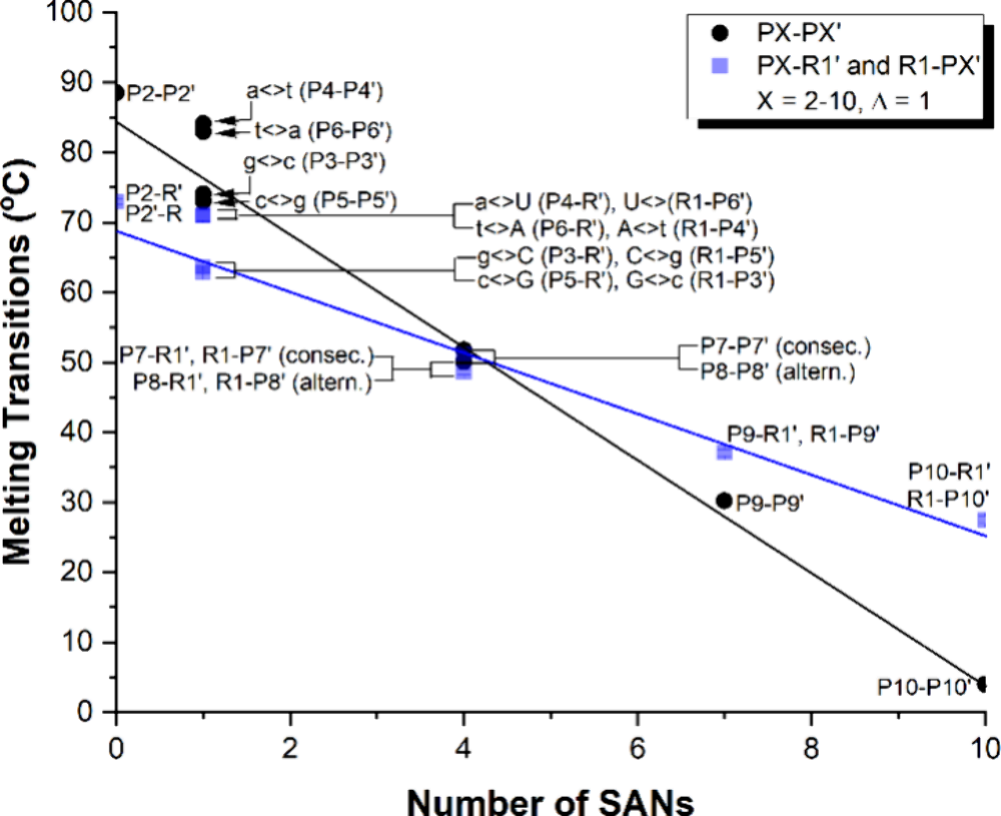
Effects of SANs on thermal stability of γPNA-γPNA (black line) and γPNA-RNA (blue line) duplexes. Filled circles and squares represent experimental data points, while solid lines indicate linear regressions. The T_m_ of the R-R’ duplex is 58.2 °C (data not shown).

### γPNA-γPNA Duplexes Exhibited Enhanced Sensitivity to SAN-Induced Destabilization Compared to γPNA-RNA

γPNA-γPNA duplexes exhibited significantly greater destabilization than γPNA-RNA duplexes, with a more pronounced effect for c-g/g-c pairs compared to a-t/t-a. This destabilization follows a linear trend: although γPNA-γPNA duplexes initially displayed significantly higher T_m_ values than γPNA-RNA, their thermal stability declined sharply as more SAN units were incorporated (Figure 3). Notably, introduction of four SAN units caused the T_m_ values of γPNA-γPNA duplexes to converge with those of γPNA-RNA. Interestingly, the placement of SANS, whether consecutive or alternating, had minimal impact on duplex thermal stability. Beyond this threshold, γPNA-γPNA duplexes became less stable than their γPNA-RNA counterparts, as observed in P9–P9’ and P10–P10’. The fully modified P10-P10’ duplex exhibited a barely detectable melting transition (∼5 °C). Although γPNA-RNA has a lower T_m_ than RNA-RNA, SAMRS-γPNA can still bind the duplex by invading both RNA strands via double-duplex invasion, with added binding free energy coming from the second strand. These trends were corroborated by electrophoretic mobility shift assays (EMSAs), which demonstrated that most probes retained the ability to bind their complementary RNA strand (Figure S25a), as well as the RNA duplex (Figure S25b), with the exception of P9 and P10, bearing 70% and 100% SAN substitutions, respectively. This finding is particularly noteworthy, as it supports our design rationale: preventing self-hybridization despite the sequence complementarity among the probes, while maintaining selective binding to RNA targets.

### Pseudoknot Target Selection and Probe Design

Pseudoknots are among the most prevalent, biologically significant, and structurally challenging RNA motifs to target.^25^ They are widely distributed across viruses, bacteria, and eukaryotic cells, including telomerase RNA, where they play essential roles in gene expression, viral infection, and telomere maintenance.^26,27^ While multiple pseudoknot folding topologies exist, the best characterized is the H-type pseudoknot (Figure 1). Its formation begins with base-pairing that creates a stem-loop structure (Step 1), followed by additional base-pairing between the loop and a neighboring region (Step 2). This sequential folding process results in a highly complex and thermodynamically stable structure composed of two stems and two loops. As such, this motif presents a stringent test for our newly developed SAMRS-γPNA system.

We selected the *Plasmodium falciparum* pseudoknot as a model for investigation due to its relevance to healthcare.^27–29^ Figure 4a depicts the pseudoknot (T1) of this malaria-causing pathogen, alongside a simplified stem-loop variant T2 (Figure 4b). We designed four probes (Figure 4c): the first three—MB1, MB2, and MB3—were fully modified with SANs and engineered to bind the *a’c*, *a’cb*, and *a’cba* regions of T1 and T2. The fourth probe, MB4, targeted the same regions as MB3 but incorporated natural nucleobases in the *c’*-domain to enhance binding affinity to the target.

**Figure 4:**
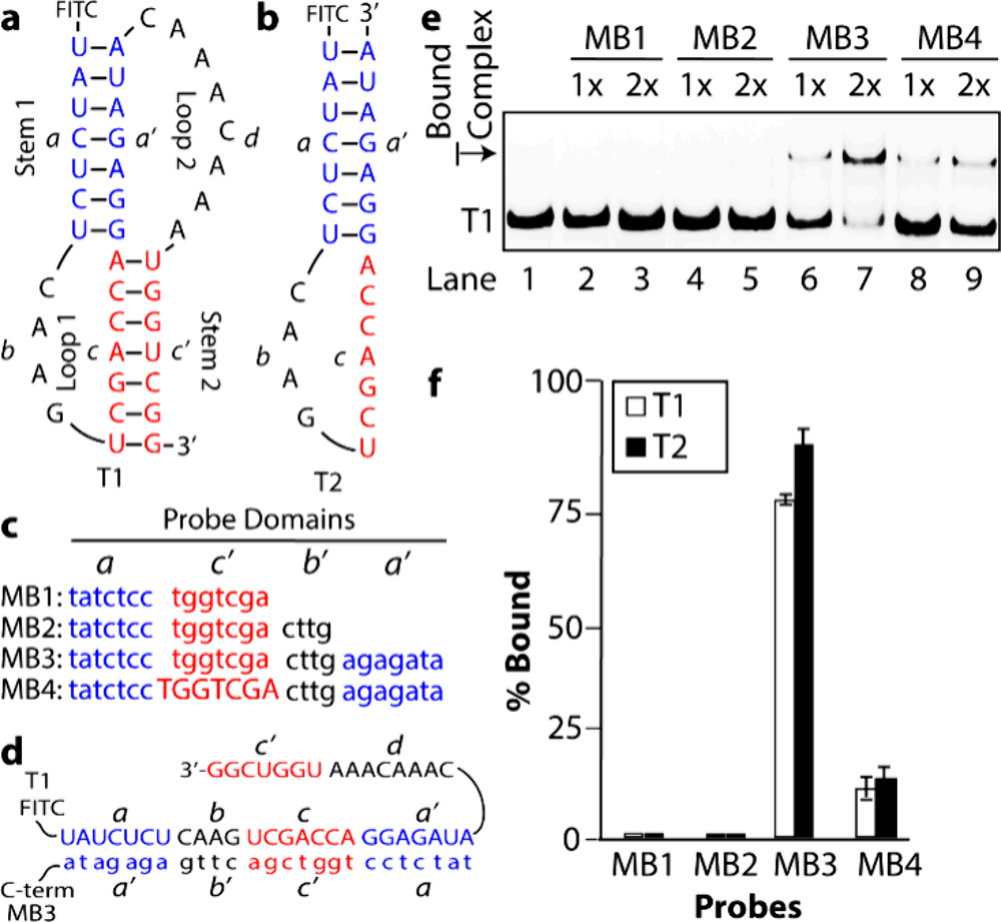
Model RNA targets and probe binding coverage. (**a**) Pseudoknot (T1) and (**b**) a simplified stem-loop variant T2. (**c**) γPNA probes designed to bind specific regions of T1 and T2; they are written from N- to C-terminus. (**d**) Representative binding of MB3 to T1 to form a duplex, with RNA domains dc’ flipped out. (**e**) ESMA results of probes binding to T1, and (**f**) quantification of probe binding to T1 and T2. Samples were prepared by mixing preannealed RNA with probes and incubated at 37 °C for 1 h in a physiologically relevant buffer (10 mM NaPi, 137 mM NaCl, 150 mM KCl, 2 mM MgCl_2_; pH 7.4) prior to separation by nondenaturing PAGE. The RNA concentration was 0.1 μM, and probe concentrations were 0.1 μM (1x) and 0.2 μM (2x).

### EMSA Analysis of Probe Binding to RNA Targets

The binding interactions between the probes and RNA targets T1 and T2 were analyzed using EMSAs. Only the probes designed to bind the full stem-loop region, MB3 and MB4, showed binding to T1 (Figure 4e, lanes 6-9), as indicated by the formation of a shifted band. Interestingly, MB4 exhibited lower binding efficiency than MB3, which was unexpected given the inclusion of natural nucleobases in *c’*-domain which was supposed to bind to its target more effectively. A similar binding pattern was observed with T2 (Figure S26); however, the appearance of an additional band, corresponding to an intermolecular T2-T2 duplex—as verified by concentration-dependent analysis and variations in the rate of cooling (Figure S27), complicated the interpretation. Based on this observation, all subsequent investigations were focused on T1, which more closely mimics the structure of the *Plasmodium falciparum* pseudoknot.

### Improving the Binding Affinity of MB3

One possible explanation for MB4’s lower binding efficiency compared to MB3 is the formation of intermolecular duplexes, similar to those observed with T2. The inclusion of natural nucleobases—intended to enhance MB4’s binding affinity to T1—may have instead promoted self-hybridization, resulting in the formation of a stable duplex structure referred to as a “Stabilizing” region (Figure 5a). This intermolecular duplex is expected to be thermodynamically more stable in MB4 than in MB3 due the presence of natural nucleobases in this region. If this hypothesis is correct, selectively replacing SANs in the bulged regions of MB3 with natural nucleobases that do not participate in intermolecular duplex formation, but still allow binding to the intended RNA target as depicted in **MB5** (Figure 5b), should enhance binding efficiency. Consistent with this prediction, EMSA data revealed that MB5 exhibited complete binding at a 1:1 probe-to-target ratio, whereas MB3 achieved only about 30% occupancy (Figure 5c, lane 6 vs lane 3). This result highlights the complexity of nucleic acid hybridization and the delicate balance between intramolecular and intermolecular interactions in stem-loop RNA structures. It also underscores the design flexibility of the newly developed SAMRS-γPNA in overcoming these challenges.

**Figure 5:**
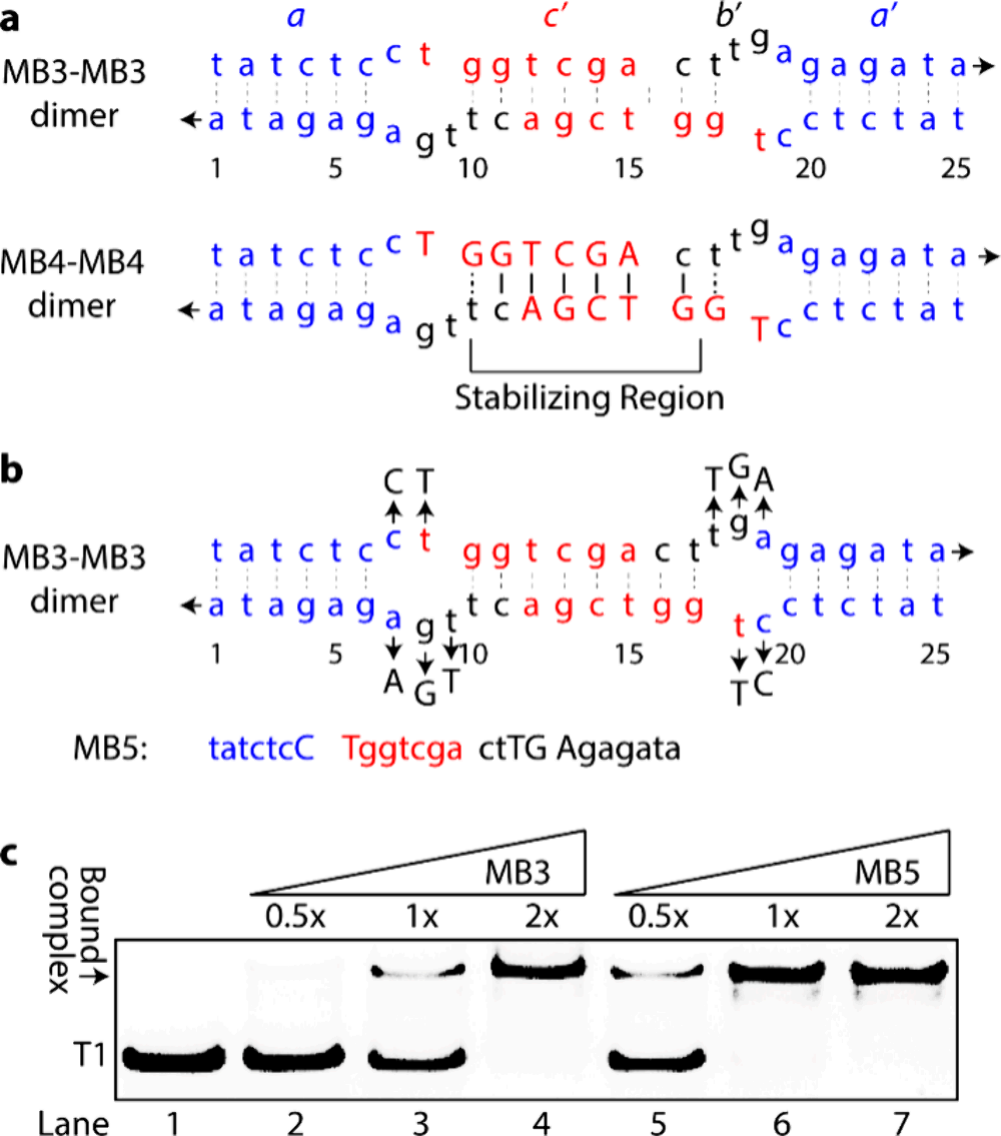
Optimization of MB3 binding. (**a**) Representations of MB3-MB3 and MB4-MB4 dimers. (**b**) Natural nucleobase substitutions to enhance MB3 binding, leading to the development of MB5. (**c**) Comparison of relative binding efficiency of MB3 and MB5. The RNA concentration was 0.1 μM, and probe concentrations were 0.05, 0.1, and 0.2 μM.

### Evaluation of MB5 Binding Sequence Specificity

MB5 is unique in that it is designed to bind the entire stem-loop region of T1 (*a’cba*-domains). SAMRS-γPNAs are expected to be more sensitive to base mismatches due to their relatively weak binding affinity and design approach, which targets the stem region where intramolecular RNA base-pairing competes with intermolecular probe hybridization. To evaluate this capability, we created three mutations in the parent T1: T3 (C4-G22→G4-C22 transversion), T4 (A10→U10 point mutation), and T5 (A15-U37→U15-A37 transversion) (Figure 6a). These mutations were designed to maintain the thermodynamic stability of T1 while introducing mismatches in key regions upon hybridization with the probes: the *a-a’* stem (T3), *b*-loop (T4), and *c*-*c*’ stem (T5). As expected, MB5 exhibited little to no binding with mismatched targets (Figure 6b, Figure S28). This finding highlights the benefit of SAMRS-γPNA in targeting both strands of the RNA stem, offering greater specificity and selectivity.

**Figure 6:**
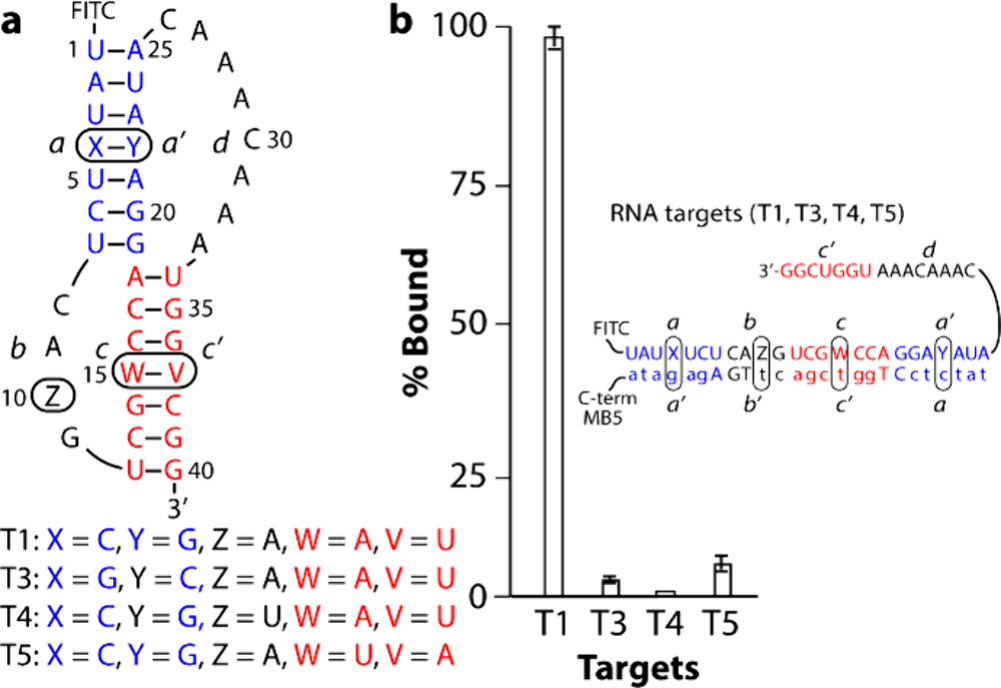
Effects of RNA mismatches on MB5 binding. (**a**) Sequences of mismatched targets (T3, T4, and T5) as highlighted in the boxes. T3 contained a C4-G22→G4-C22 transversion in domains *a* and *a’*. Hybridization of MB5 to T3 creates two mismatches, while MB5 to T4 and T5 induces a single mismatch. (**b**) Comparison of binding efficiency. *Inset*: hybridization of MB5 to the various RNA targets to produce perfectly matched (MB5-T1) and mismatched (MB5-T3, MB5-T4, and MB5-T5) duplexes.

### Benchmarking MB5′s Specificity against Other Nucleic Acid Systems

To better understand MB5’s specificity compared to other nucleic acid systems, we assessed its binding efficiency against DNA (D1), Morpholino (M1), RNA (R1), and interspersed RNA/LNA (L1) probes (Figure 7a). Each probe, 18 units in length, was designed to target the *a’cb*-region of T1 and T3, sharing the same sequence but different in binding strength (Figure 7b and 7c). We originally did not include full-length (25-mer) probes like MB5, as those containing natural nucleobases were predicted to self-hybridize rather than bind the intended target. This prediction was later confirmed experimentally (data not shown), which supports our decision to focus the comparison on the 18-mer probes. Among them, L1, in which LNA units were inserted into the RNA strand at every third position, exhibited the strongest binding affinity due to its conformationally matched, rigid backbone. Unlike MB5, these probes excluded the *a’*-domain to prevent self-hybridization, which could interfere with target binding. Inspection of Figure 7d (*Panel A*) showed that MB5 achieved complete binding with the perfectly matched target (T1) (lane 2), whereas only partial binding was observed with the remaining probes (lanes 3 through 6). The latter outcome was unexpected, particularly for L1, which was anticipated to exhibit the strongest binding within this group. One possible explanation is the formation of secondary structures that may have interfered with their binding.

**Figure 7:**
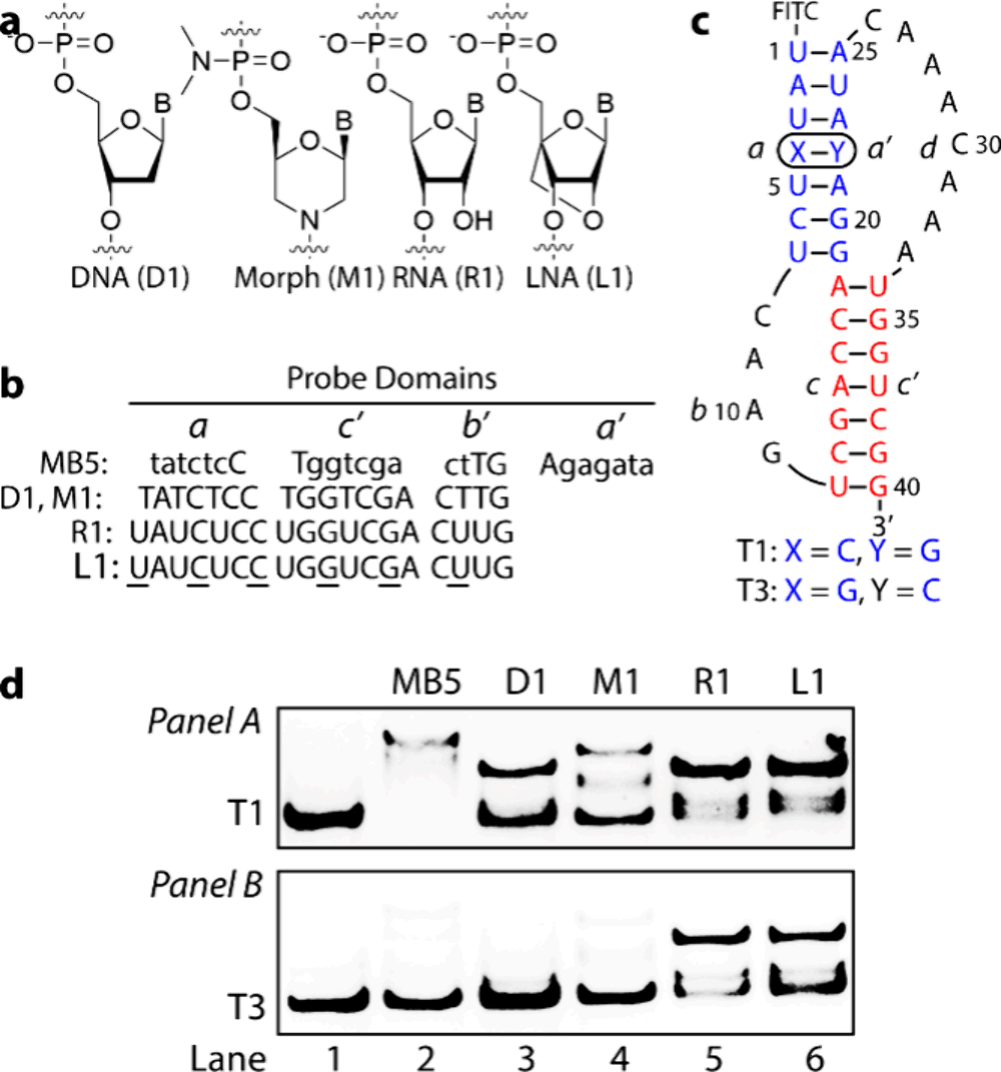
Comparison of binding efficiency and specificity of MB5 to DNA (D1), Morpholino (M1), RNA (R1), and RNA/LNA (L1). (**a**) Chemical structures of D1, M1, R1, and L1. (**b**) The sequence of MB5, D1, M1, R1, and L1 probes (LNA units are underlined). (**c**) T1 and T3 RNA targets. (**d**) Comparison of binding efficiency (*Panel A*) and specificity (*Panel B*) of MB5 to the other nucleic acid systems.

Compared with the mismatch T3 (Figure 7c, *Panel B*), binding was completely inhibited for MB5 (lane 2), D1 (lane 3), and M1 (lane 4) probes. This was expected, as D1 and M1 are relatively weak binders and had exhibited only partial binding even with the perfectly matched target. In contrast, the binding patterns of R1 and L1 closely resembled those observed with T1 (lanes 5 and 6, *Panel A* vs *Panel B*), *albeit* with slightly lower efficiency, as indicated by the intensity of the shifted bands. These results align with reported observations,^30^ where weak binders tend to be more sequence specific but less effective in targeting structured RNA, as demonstrated by DNA and Morpholino. On the other hand, while the nonspecific binding of R1 and especially L1, was anticipated due to their high affinity, their incomplete binding with the perfect-match T1 was unexpected. Equally intriguing was the presence of two shifted bands for M1, R1, and L1 (*Panel A*, lanes 3–6), suggesting the formation of two stable complexes. It is important to note that in these EMSA measurements, the targets (T1 and T3) were fluorescently labeled, meaning that only RNA-bound probes appeared on the gel, while unbound probes remained undetected. We acknowledge that this is not a direct comparison, as these oligonucleotides did not contain the same SAN sequence as MB5. Nonetheless, it provides a broad overview of the two systems, strand invasion and double-duplex invasion, with respect to recognition specificity.

Several factors could contribute to these observations. The incomplete binding of D1, M1, R1, and L1 with T1 (Figure S29a), especially the last two, could result from probe self-sequestration through dimer formation (Figure S29b). This hypothesis is supported by findings from MB4 binding studies (Figure 5a). The higher the affinity, the more likely they self-hybridize. The presence of two stable complexes in the gel likely arises from two distinct hybridization pathways. The first involves hybridization of probes to the target, resulting in the expected hybrid duplex (Figure S29c, COMPLEX 1). The second stems from the cascade hybridization chain reaction (HCR),^31^ where probe binding to T1 generates two toeholds (Toehold 1 and Toehold 2). These toeholds catalyze the hybridization of additional T1 molecules, creating new toeholds (Toehold 3 and Toehold 4) and ultimately leading to the formation of a stable T1-T1 dimer (Figure S29c, COMPLEX 2). This interpretation is supported by the results from T2 folding characterizations (Figure S26, lane 1), which revealed two stable states: a hairpin and a dimer. Once formed, the dimer is highly stable and more resistant to disruption than the hairpins.

## Discussion

Antisense technologies offer several advantages over small-molecule approaches for targeting nucleic acids, including broad sequence recognition and high specificity. However, a major challenge lies in balancing binding efficiency and specificity, especially when targeting complex RNA structures common in their native states. Figure 8 depicts various probe hybridization scenarios. Weak-binding probes, such as DNA and morpholinos, often lack sufficient binding free energy to outcompete RNA folding when targeting only one side of the stem and loop (Figure 8a, Probe 1). Extending the probe to cover both sides of the stem (Probe 2) is counterproductive, as it tends to self-hybridize and form its own secondary structure. In contrast, high-affinity probes, such as LNA, PNA, and γPNA composed of natural nucleobases (Figure 8b, Probe 3), can outcompete RNA folding barriers. However, their strong binding increases the risk of off-target effects and toxicity. In addition to binding perfect-matches, these probes can also tolerate mismatches, insertions, and deletions, which may reduce overall specificity.

**Figure 8:**
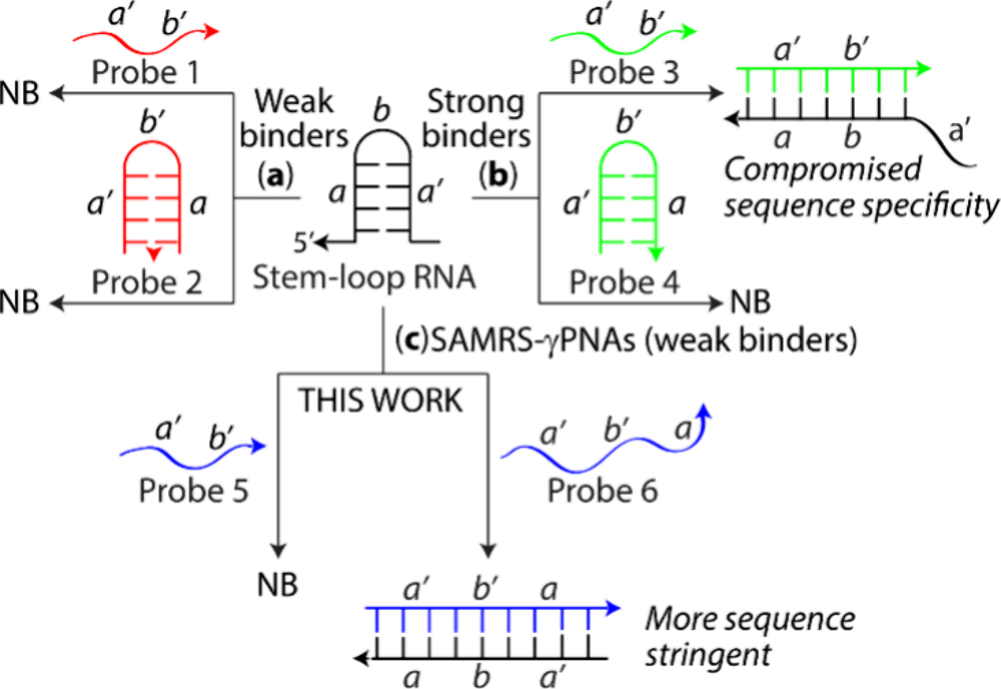
Hybridization of nucleic acid probes to a stem-loop RNA target. (**a**) Weak binders, (**b**) strong binders, and (**c**) SAMRS-γPNAs (weak binders). NB: No binding.

To mitigate these challenges, antisense probes are typically designed to target unstructured RNA regions using tiling or combinatorial approaches. While this had led to some success, highlighted by recent FDA-approval of antisense drugs,^32^ these empirical approaches lacks strong design rationale and are difficult to standardize. In this work, we directly address the challenge of targeting structured RNA through the development of SAMRS-γPNAs. These probes generally have lower affinity for RNA than their natural DNA or RNA counterparts. When designed to bind one side of the stem and loop (Figure 8c, Probe 5), SAMRS-γPNAs are unable to effectively engage the target. However, when extended to bind both sides of the stem, binding becomes more effective and highly sequence specific, in contrast with the existing nucleic acid systems containing natural nucleobases. The added binding free energy comes from hybridization with both arms of the RNA target.

This targeting strategy favors structured motifs, such as stem-loops, over unstructured regions due to their higher thermodynamic stability. Moreover, it enhances sequence specificity, as even a single mismatch, insertion, or deletion in the stem region disrupts binding across both arms of the probe (Figure 8c, Probe 6). While the work is still in its nascent stages, it demonstrates that targeting both sides of the RNA stem-loop regions can overcome current limitations of probe design for native RNA structures. Further improvements are needed, including the development of SANs that enhance RNA binding while maintaining, or further destabilizing, γPNA-γPNA duplexes. One such example is the replacement of c and a with clamp-G and 2,6-diaminopurine, respectively. Another area involves expanding the aromatic ring systems to improve base-stacking, compensating for the loss of hydrogen-bonding interactions.

## Conclusion

In summary, we have demonstrated, as a proof-of-concept, that when specifically designed SANs (a, c, g, and t) are incorporated into the conformationally preorganized γPNA backbone, they can be employed to target the full RNA stem-loop with high specificity and selectivity. This targeting strategy offers both design flexibility and recognition precision, leveraging the γPNA backbone to fine-tune region-specific binding affinity through a combination of SAN, natural, and modified nucleobase substitutions. As a critical first step toward a more general and effective RNA-targeting strategy, this work holds promise for biological research and therapeutic development.

## Supplementary Material



## Data Availability

The data underlying this study are available in the published article and its Supporting Information

## Notes

Nucleobase and monomer synthesis, along with the NMR and MS spectra (PDF)
