# Supplementary material for: Self-Avoiding Gamma Peptide Nucleic Acids for Selective Targeting of RNA Secondary Structures
Source: ACS Chem Biol. 2026 Mar 17;21(4):666–73. doi: 10.1021/acschembio.5c00830 (PMC13097139; doi:10.1021/acschembio.5c00830)
Supplement: Supplementary file 1 [file cb5c00830_si_001.pdf]

SUPPLEMENTAL INFORMATION

Self-Avoiding Gamma Peptide Nucleic Acids for Selective Targeting of RNA Secondary Structures

Isha Dhami,† Shivaji A. Thadke,† J. Dinithi R. Perera, Ramesh Batwal, En Zheng, Savani W. Thrikawala, Babita Aryal, and Danith H. Ly*

Department of Chemistry and Institute for Biomolecular Design and Discovery (IBD), Carnegie Mellon University, 4400 Fifth Avenue, Pittsburgh, Pennsylvania 15213, United States.

Email: dly@andrew.cmu.edu

Table of Contents **Page**

General Techniques		3
**Figure S1**	Hydrogen-bonding patterns of c-g, X-g, c-G, and X-G	4
**Figure S2**	RP-HPLC chromatograms and MALDI-TOF spectra of P1	5
**Figure S3**	RP-HPLC chromatograms and MALDI-TOF spectra of P1’	5
**Figure S4**	RP-HPLC chromatograms and MALDI-TOF spectra of P2	6
**Figure S5**	RP-HPLC chromatograms and MALDI-TOF spectra of P2’	6
**Figure S6**	RP-HPLC chromatograms and MALDI-TOF spectra of P3	7
**Figure S7**	RP-HPLC chromatograms and MALDI-TOF spectra of P3’	7
**Figure S8**	RP-HPLC chromatograms and MALDI-TOF spectra of P4	8
**Figure S9**	RP-HPLC chromatograms and MALDI-TOF spectra of P4’	8
**Figure S10**	RP-HPLC chromatograms and MALDI-TOF spectra of P5	9
**Figure S11**	RP-HPLC chromatograms and MALDI-TOF spectra of P5’	9
**Figure S12**	RP-HPLC chromatograms and MALDI-TOF spectra of P6	10
**Figure S13**	RP-HPLC chromatograms and MALDI-TOF spectra of P6’	10
**Figure S14**	RP-HPLC chromatograms and MALDI-TOF spectra of P7	11
**Figure S15**	RP-HPLC chromatograms and MALDI-TOF spectra of P7’	11
**Figure S16**	RP-HPLC chromatograms and MALDI-TOF spectra of P8	12
**Figure S17**	RP-HPLC chromatograms and MALDI-TOF spectra of P8’	12
**Figure S18**	RP-HPLC chromatograms and MALDI-TOF spectra of P9	13
**Figure S19**	RP-HPLC chromatograms and MALDI-TOF spectra of P9’	13
**Figure S20**	RP-HPLC chromatograms and MALDI-TOF spectra of P10	14
**Figure S21**	RP-HPLC chromatograms and MALDI-TOF spectra of P10’	14
**Figure S22**	UV-melting profiles of γPNA-γPNA duplexes containing various number of SAN substitutions.	15
**Figure S23**	UV-melting profiles of Series 1 RNA-γPNA duplexes containing various number of SAN substitutions	16
**Figure S24**	UV-melting profiles of Series 2 RNA-γPNA duplexes containing various number of SAN substitutions	17
**Figure S25**	Results of EMSA binding studies	18
**Figure S26**	Results of EMSA binding studies with T2	19
**Figure S27**	Analysis of the conformation of T2	20
**Figure S28**	The effects of single-base RNA mismatches on MB5 binding	21
**Figure S29**	Probe hybridization pathways	22
**Figure S30**	Spectroscopic data for Compound 1a	23, 24
**Figure S31**	Spectroscopic data for Compound 1b	25, 26
**Figure S32**	Spectroscopic data for Compound 1c	27, 28
**Figure S33**	Spectroscopic data for Compound 1d	29, 30

**Figure S34**	Spectroscopic data for Modified a$^{(Ac)}$ Nucleobase	31, 32
**Figure S35**	Spectroscopic data for Modified g Nucleobase	33, 34
Experimental Procedures		35-38

General Techniques

All chemicals and starting materials were purchased commercially and used as received. Reactions were carried out under a nitrogen atmosphere using dried solvents, and products—whether solids, syrups, or liquids—were dried under high vacuum. Analytical TLC was performed on precoated silica gel plates (60F-254, 0.25 mm) with visualization achieved via UV light or ninhydrin/iodine staining. NMR spectra were recorded on a 500 MHz Bruker instrument using $CDCl_3$, DMSO, or D_2O with TMS as the internal standard, with multiplicities abbreviated as follows: s (singlet), d (doublet), t (triplet), q (quartet), m (multiplet), ddt (doublet of doublet of triplet), dt (doublet of triplet), td (triplet of doublet), ABq (AB quartet), dd (doublet of doublet), tt (triplet of triplet), and b (broad). In the supporting information, additional abbreviations include EA (ethyl acetate), DMF (dimethylformamide), Hex (hexane), MeOH (methanol), DCM (dichloromethane), Ether (diethyl ether), and IPA (isopropanol). High-resolution mass spectrometry (HRMS) was conducted using a Waters DART analyzer, while low-resolution mass spectrometry (LRMS) was performed with an ESI-Ion Trap-MS analyzer. PNA oligomers were synthesized via standard Fmoc-based solid-phase synthesis on MBHA-PAL resin (100–200 mesh) using a Gyros Protein Technologies Chorus instrument. Oligomer purification was typically achieved on a Hypersil GOLD aQ C18 Polar Endcapped HPLC column (25 cm × 4.6 mm, 5 µm) using a Shimadzu RP-HPLC system with an elution gradient of 0–40% acetonitrile (0.1% TFA) in water (0.1% TFA) at 55 °C over 40 min at a flow rate of 1.0 mL/min, with detection at 260 nm. MALDI-TOF spectra were acquired on a Bruker MALDI-TOF spectrometer using CHCA as the matrix, while UV-melting profiles were recorded at 260 nm on an Agilent Cary UV-Vis spectrophotometer at a heating rate of 1 °C/min, with the spectra smoothed using a 10-point adjacent average and melting temperatures determined from the first derivative. Electrophoretic mobility shift assays (EMSA) were performed using a Hoefer gel-electrophoresis system and imaged with a Protein Simple FluorChem M gel imaging system.

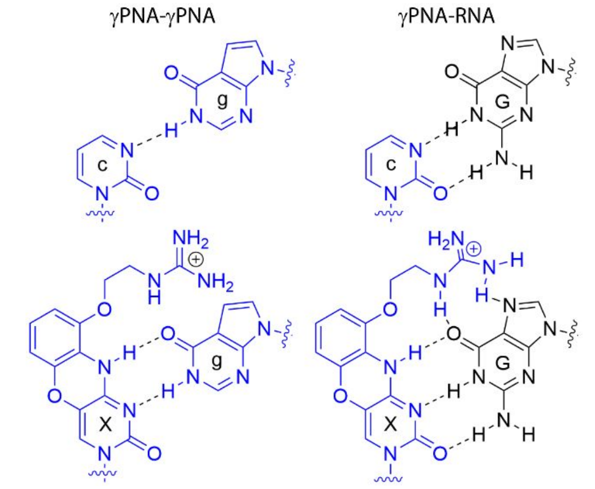

Figure S1. Hydrogen-bonding patterns of c-g, X-g, c-G, and X-G pairs.

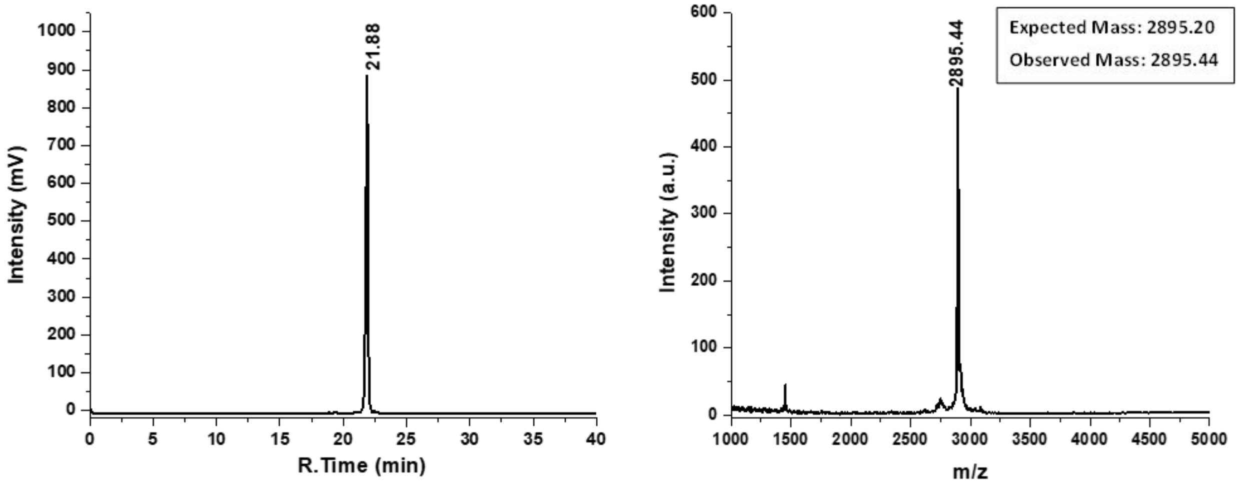

Figure S2. RP-HPLC (*left*) Gradient: 5%-40% ACN (0.1%TFA)/Water (0.1%TFA), tR=21.88 min and MALDI-TOF (*Right)* of P1 (Achiral: H-ATGGACTTCA-Lys-NH_2).

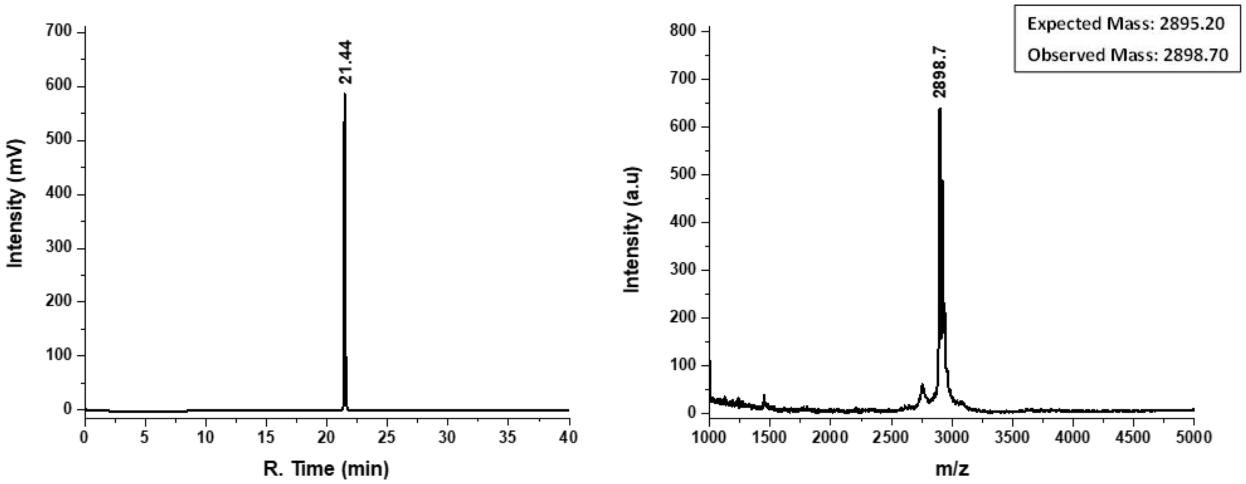

Figure S3. RP-HPLC (*left*) Gradient: 5%-40% ACN (0.1%TFA)/Water (0.1%TFA), tR=21.44 min and MALDI-TOF (*Right)* of P1' (Achiral: H-*TGAAGTCCAT*-Lys-NH_2).

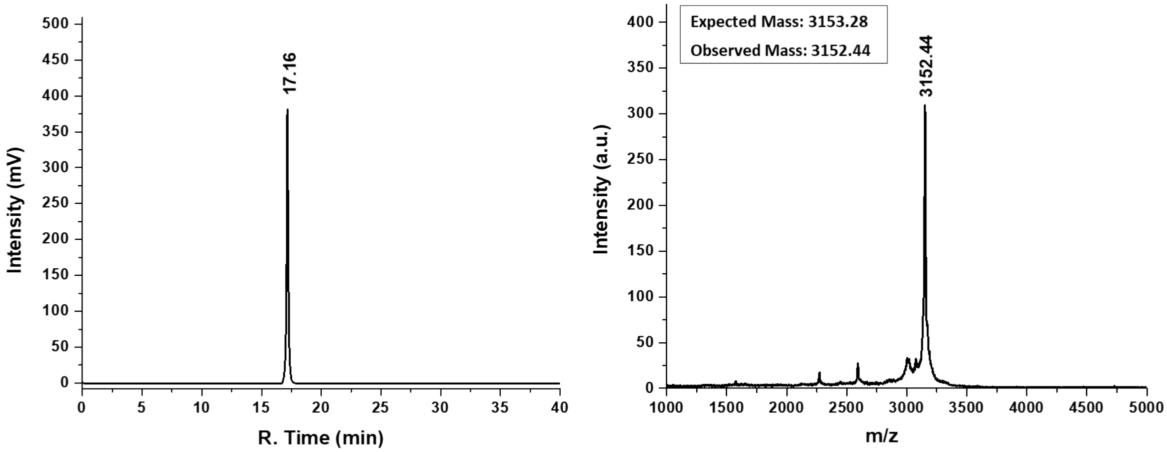

Figure S4. RP-HPLC (*left*) Gradient: 5%-40% ACN (0.1%TFA)/Water (0.1%TFA), tR=17.16 min and MALDI-TOF (*Right)* of P2 (H-ATGGACTTCA-Lys-NH_2).

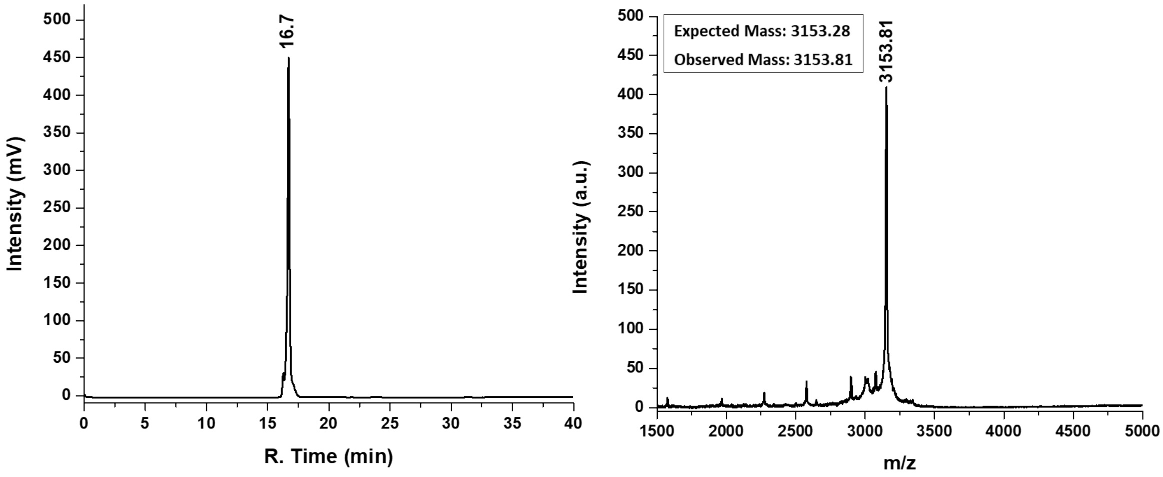

Figure S5. RP-HPLC (*left*) Gradient: 5%-40% ACN (0.1%TFA)/Water (0.1%TFA), tR=16.7 min and MALDI-TOF (*Right)* of P2' (H-TGAAGTCCAT-Lys-NH_2).

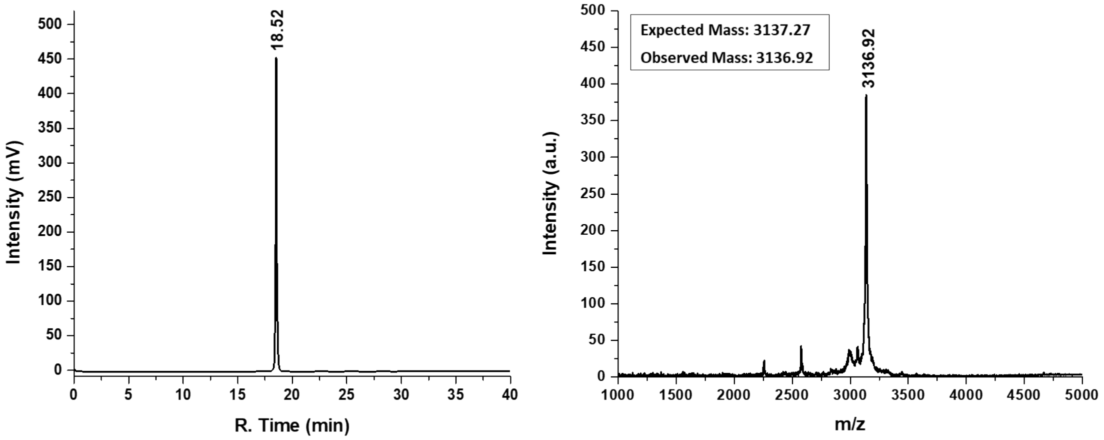

Figure S6. RP-HPLC (*left*) Gradient: 5%-40% ACN (0.1%TFA)/Water (0.1%TFA), tR=18.52 min and MALDI-TOF (*Right)* of P3 (H-ATGgACTTCA-Lys-NH_2).

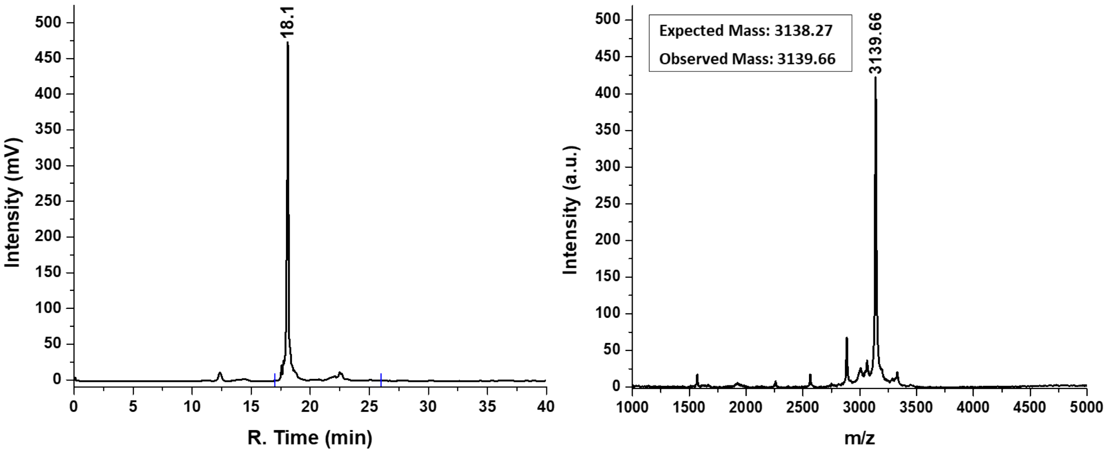

Figure S7. RP-HPLC (*left*) Gradient: 5%-40% ACN (0.1%TFA)/Water (0.1%TFA), tR=18.1 min and MALDI-TOF (*Right)* of P3' (H-TGAAGTcCAT-Lys-NH_2).

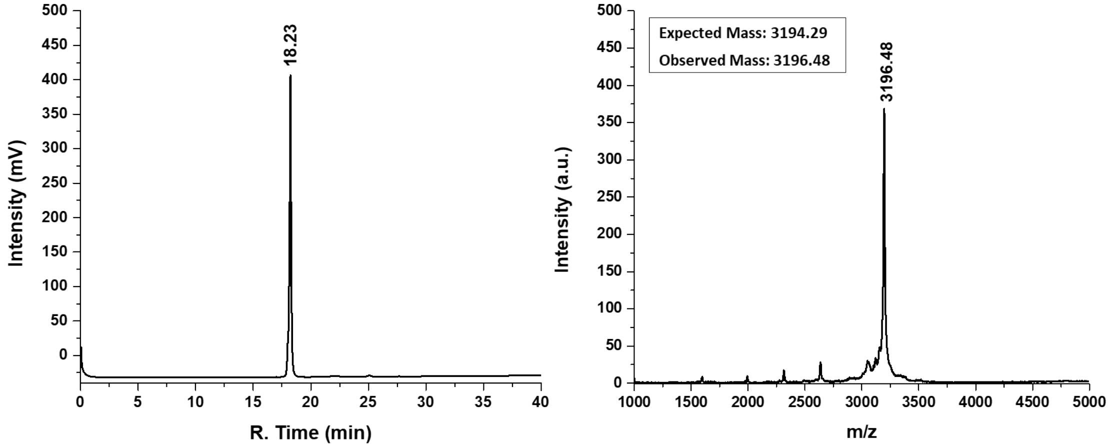

Figure S8. RP-HPLC (*left*) Gradient: 5%-40% ACN (0.1%TFA)/Water (0.1%TFA), tR=18.23 min and MALDI-TOF (*Right)* of P4 (H-ATGGaCTTCA-Lys-NH_2).

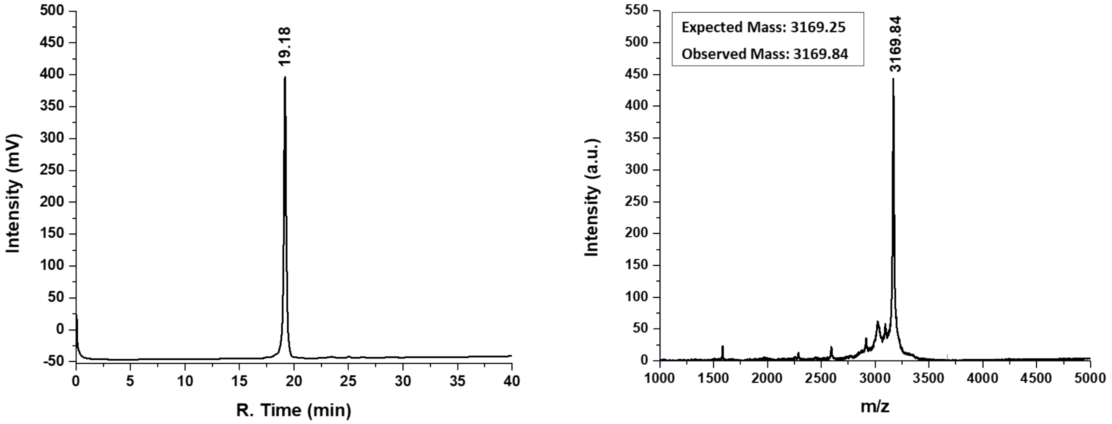

Figure S9. RP-HPLC (*left*) Gradient: 5%-40% ACN (0.1%TFA)/Water (0.1%TFA), tR=19.18 min and MALDI-TOF (*Right)* of P4' (H-TGAAGtCCAT-Lys-NH_2).

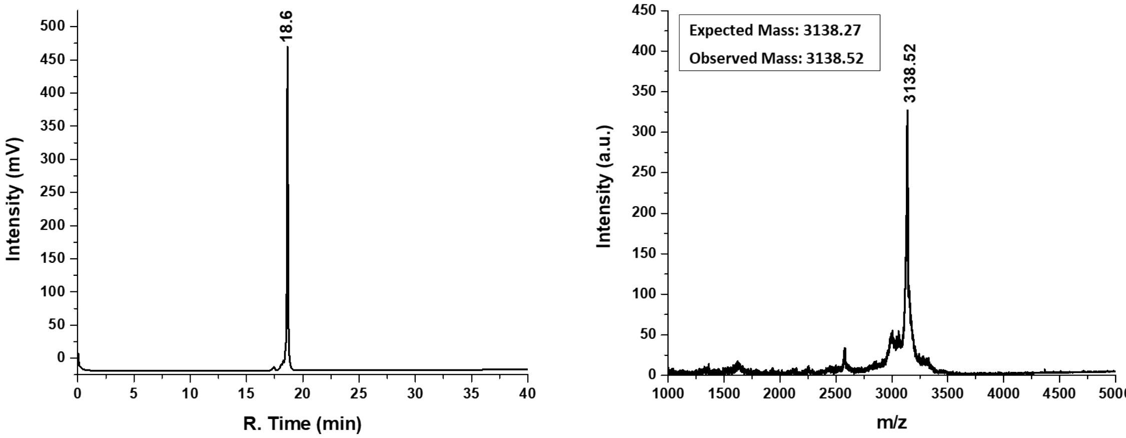

Figure S10. RP-HPLC (*left*) Gradient: 5%-40% ACN (0.1%TFA)/Water (0.1%TFA), tR=18.60 min and MALDI-TOF (*Right)* of P5 (H-ATGGcTTCA-Lys-NH_2).

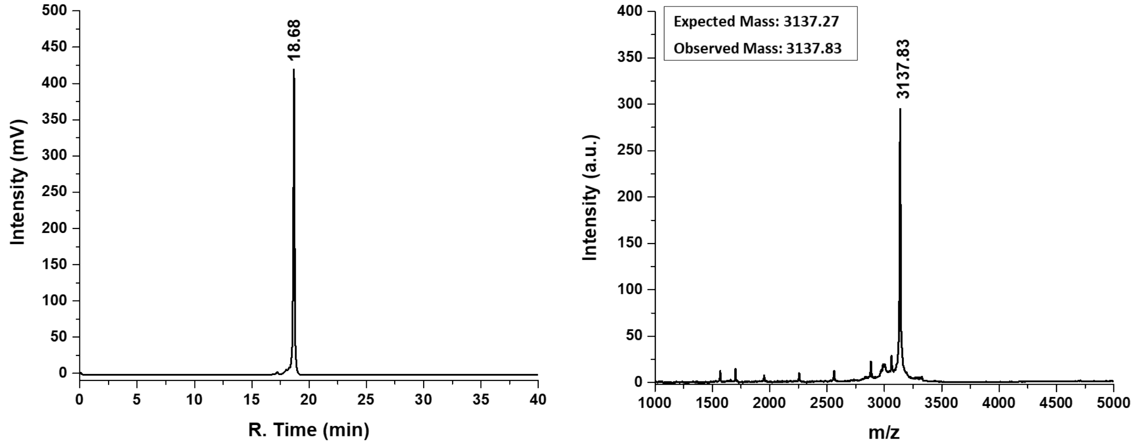

Figure S11. RP-HPLC (*left*) Gradient: 5%-40% ACN (0.1%TFA)/Water (0.1%TFA), tR=18.68 min and MALDI-TOF (*Right)* of P5' (H-TGAAgTCCAT-Lys-NH_2).

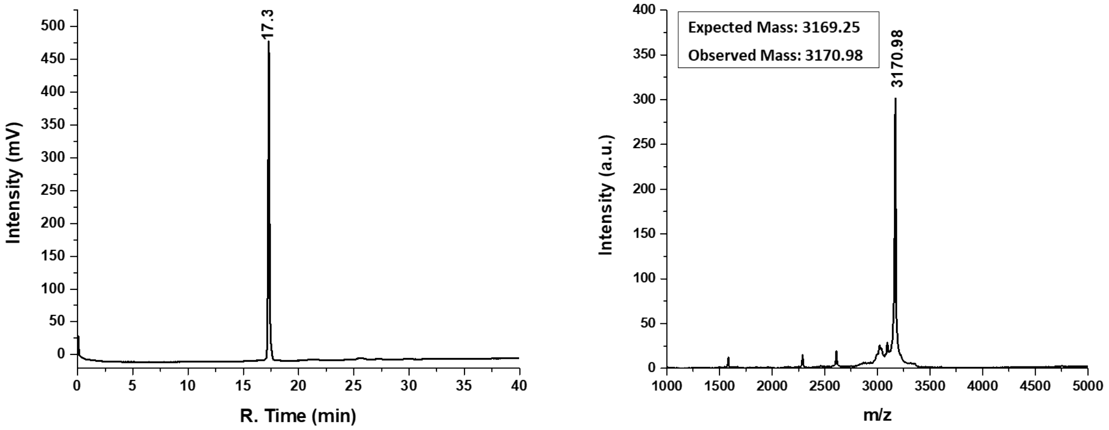

Figure S12. RP-HPLC (*left*) Gradient: 5%-40% ACN (0.1%TFA)/Water (0.1%TFA), tR=17.3 min and MALDI-TOF (*Right)* of P6 (H-ATGGACtTCA-Lys-NH_2).

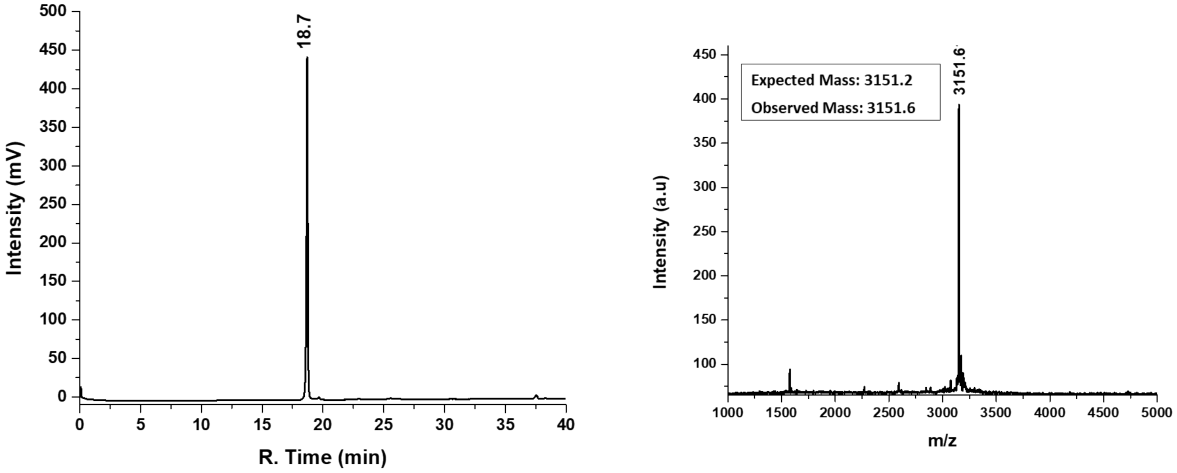

Figure S13. RP-HPLC (*left*) Gradient: 5%-40% ACN (0.1%TFA)/Water (0.1%TFA), tR=18.70 min and MALDI-TOF (*Right)* of P6' (H-TGAaGTCCAT-Lys-NH_2).

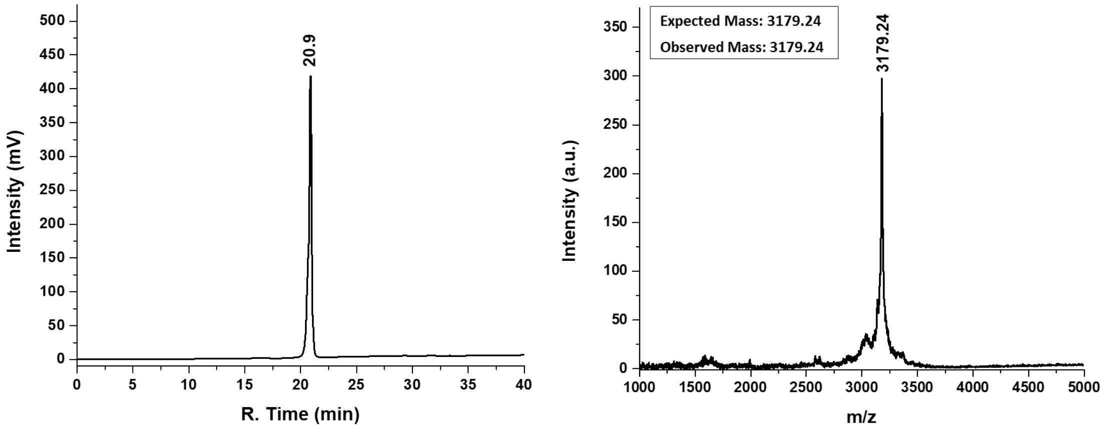

Figure S14. RP-HPLC (*left*) Gradient: 5%-40% ACN (0.1%TFA)/Water (0.1%TFA), tR=20.90 min and MALDI-TOF (*Right)* of P7 (H-ATGgactTCA-Lys-NH_2).

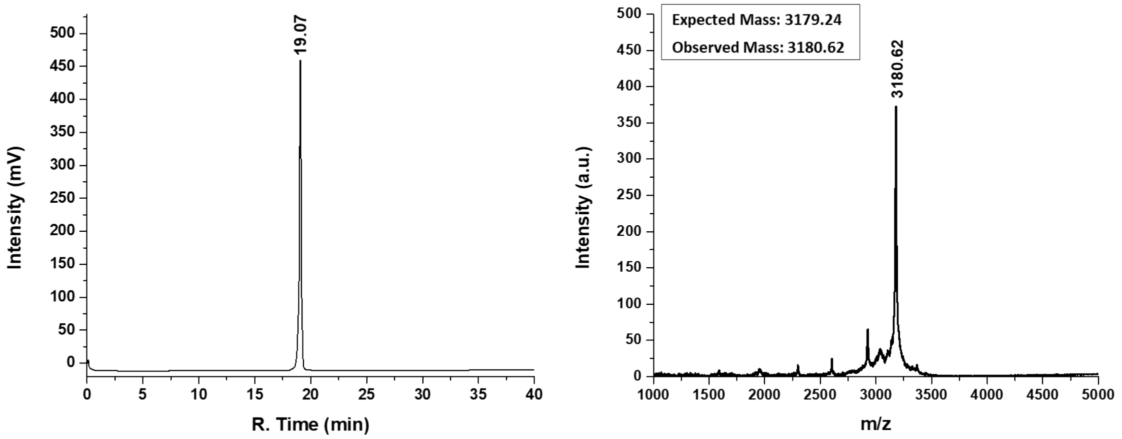

Figure S15. RP-HPLC (*left*) Gradient: 5%-40% ACN (0.1%TFA)/Water (0.1%TFA), tR=19.07 min and MALDI-TOF (*Right)* of P7’ (H-TGAagtcCAT-Lys-NH_2).

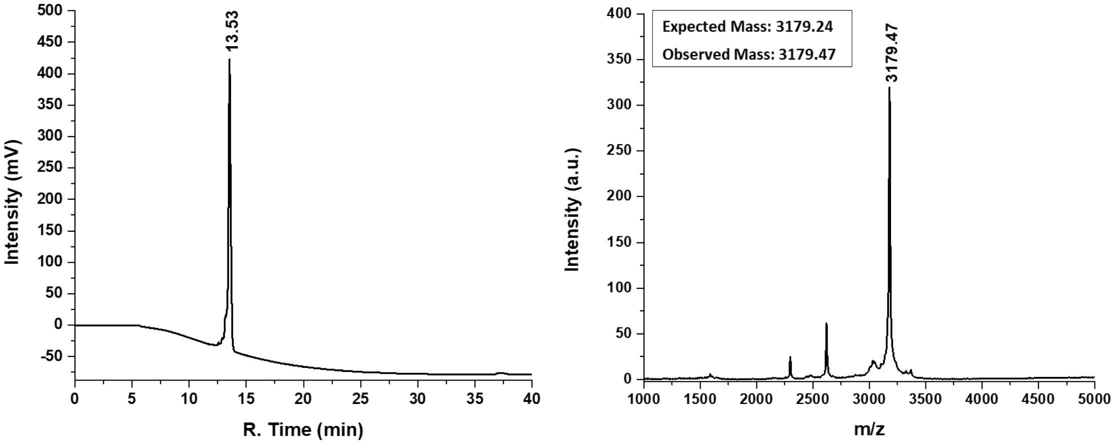

Figure S16. RP-HPLC (*left*) Gradient: 5%-40% ACN (0.1%TFA)/Water (0.1%TFA), tR=13.53 min and MALDI-TOF (*Right)* of P8 (H-ATGgAcTtCa-Lys-NH_2).

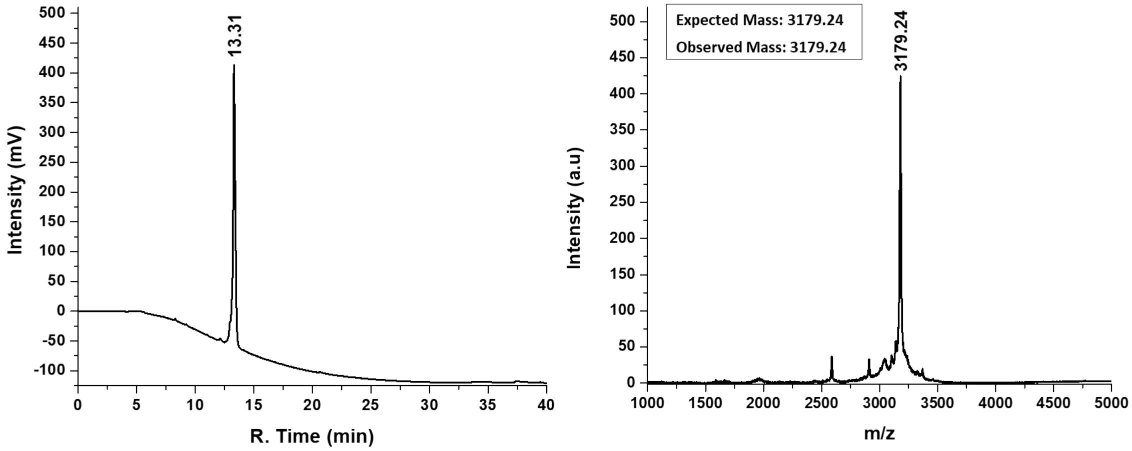

Figure S17. RP-HPLC (*left*) Gradient: 5%-40% ACN (0.1%TFA)/Water (0.1%TFA), tR=13.31 min and MALDI-TOF (*Right)* of P8' (H-tGaAgTcCAT-Lys-NH_2).

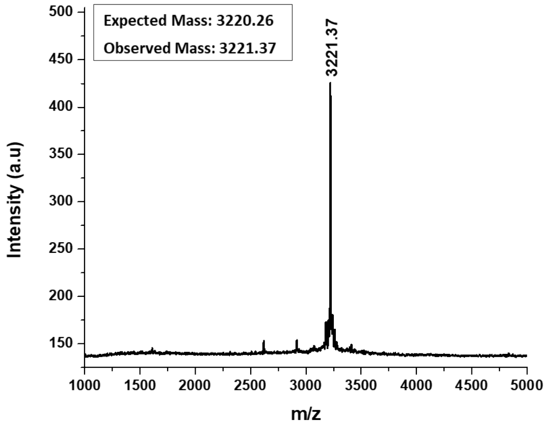

Figure S18. RP-HPLC (*left*) Gradient: 5%-40% ACN (0.1%TFA)/Water (0.1%TFA), tR=XX min and MALDI-TOF (*Right)* of P9 (H-aTggActtCa-Lys-NH_2).

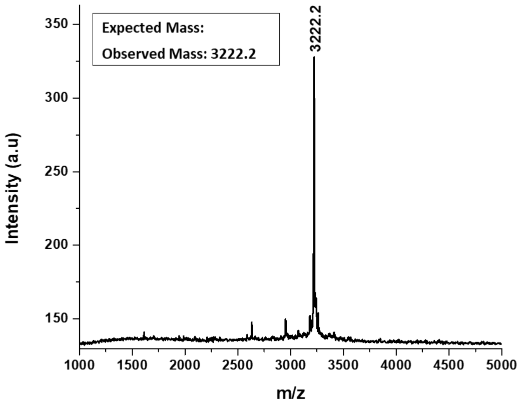

Figure S19. RP-HPLC (*left*) Gradient: 5%-40% ACN (0.1%TFA)/Water (0.1%TFA), tR=XX min and MALDI-TOF (*Right)* of P9' (H-tGaagTccAt-Lys-NH_2).

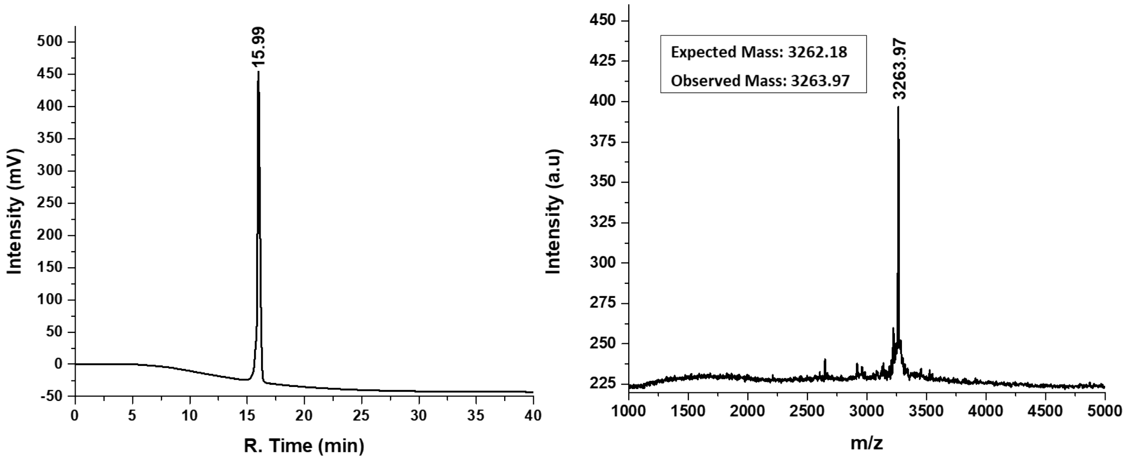

Figure S20. RP-HPLC (*left*) Gradient: 5%-40% ACN (0.1%TFA)/Water (0.1%TFA), tR=15.99 min and MALDI-TOF (*Right)* of P10 (H-atggacttca-Lys-NH_2).

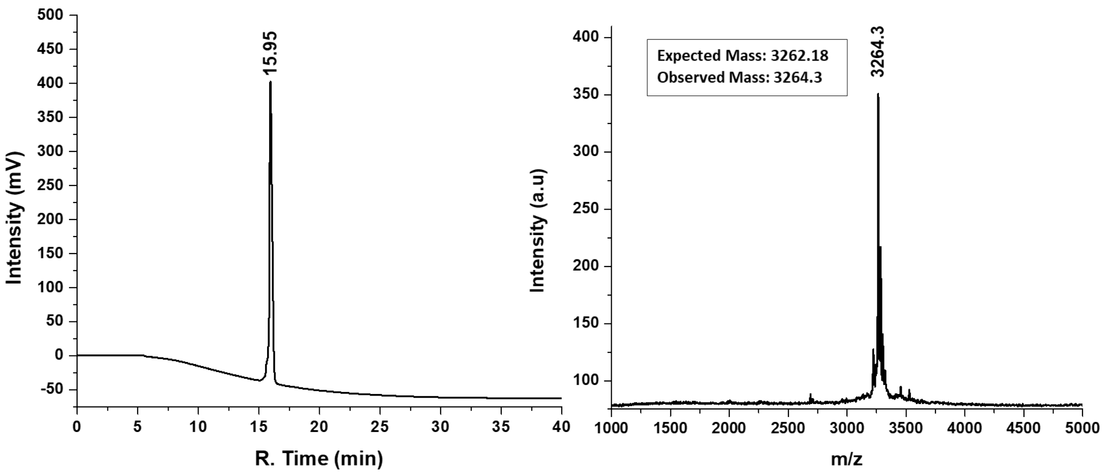

Figure S21. RP-HPLC (*left*) Gradient: 5%-40% ACN (0.1%TFA)/Water (0.1%TFA), tR=15.95 min and MALDI-TOF (*Right)* of P10' (H-tgaagtccat-Lys-NH_2).

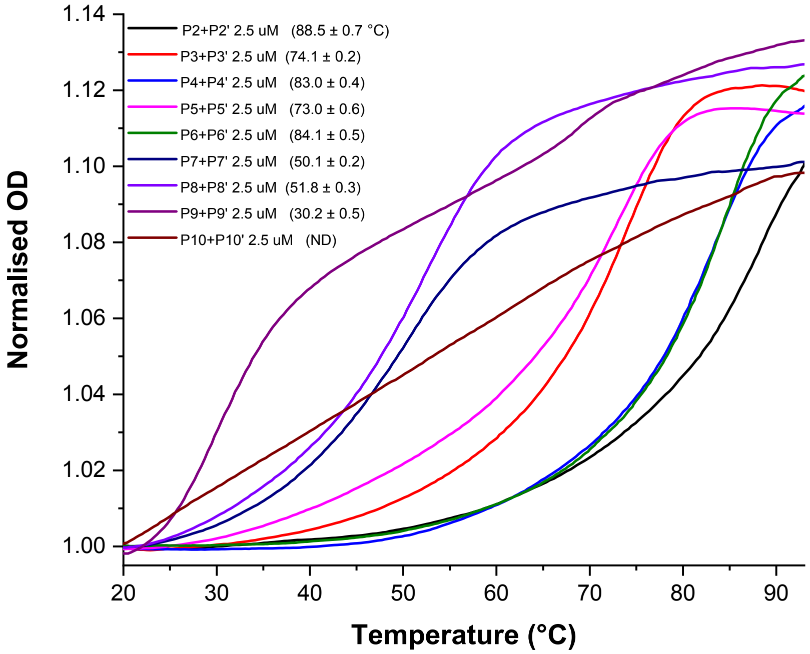

Figure S22. UV-melting profiles of γPNA-γPNA duplexes containing various number of SAN substitutions. The concentration of each strand was 2.5 μM, prepared in 10 mM NaPi buffer.

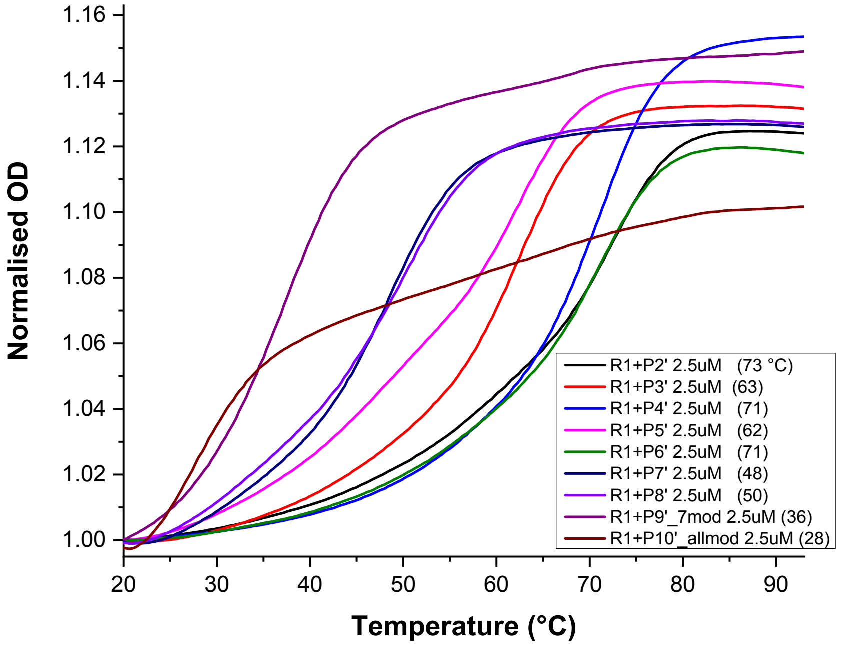

Figure S23. UV-melting profiles of Series 1 RNA-γPNA duplexes containing various number of SAN substitutions. The concentration of each strand was 2.5 μM, prepared in 10 mM NaPi buffer.

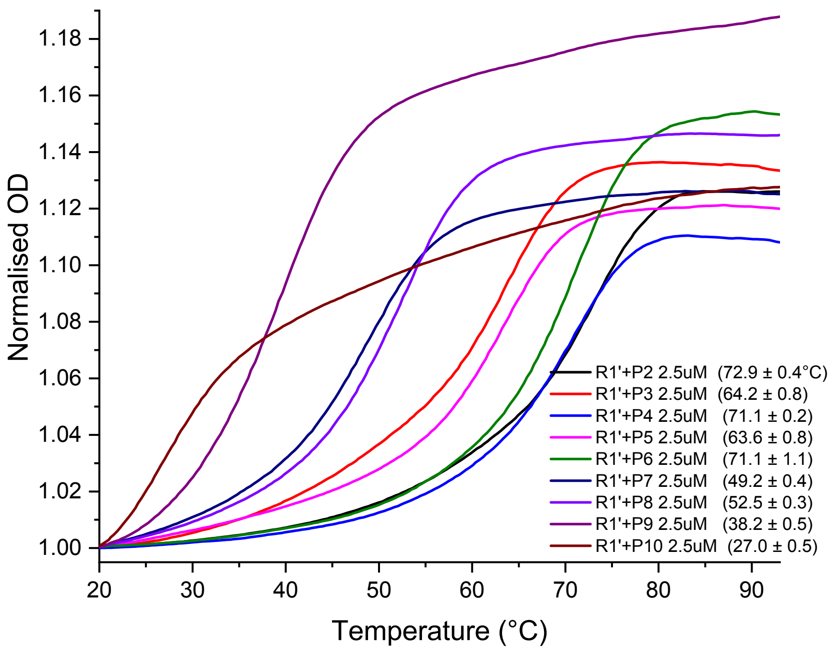

Figure S24. UV-melting profiles of Series 2 RNA-γPNA duplexes containing various number of SAN substitutions. The concentration of each strand was 2.5 µM, prepared in 10 mM NaPi buffer.

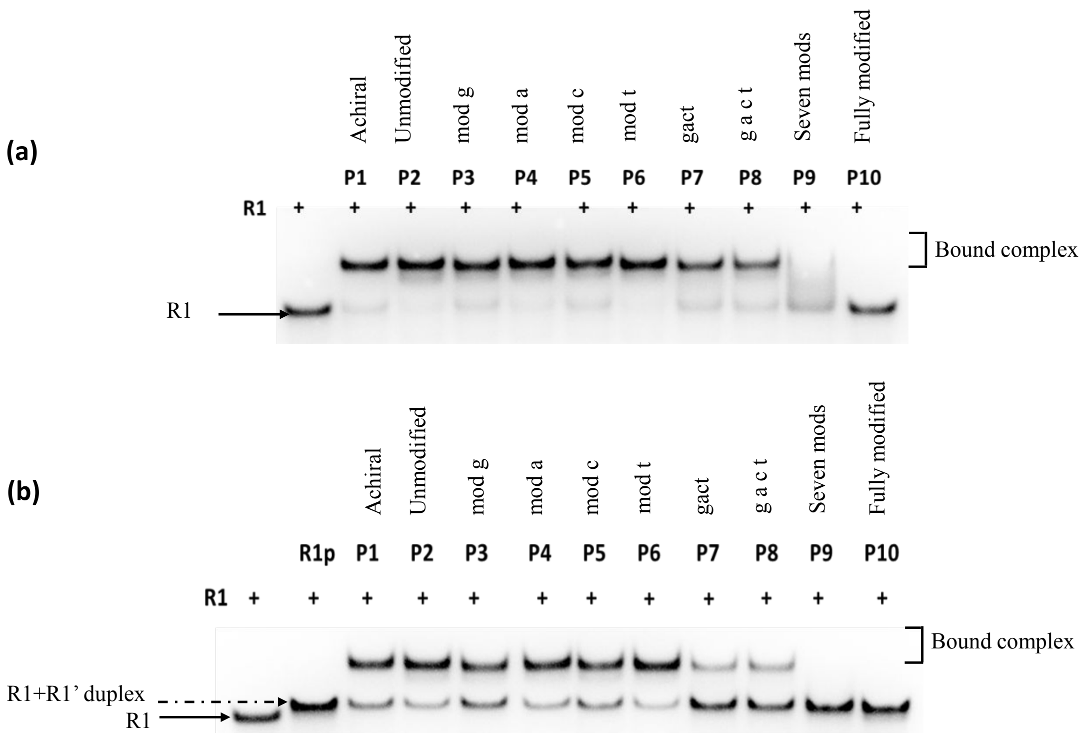

Figure S25. Results of EMSA binding studies. (**a**) With single-stranded RNA (R1), and (**b**) with RNA duple (R1-R1'). The samples were prepared by mixing pre-annealed RNA and the indicated oligomers and incubated at 37 °C for 1 h in a physiologically relevant buffer (10 mM NaPi, 137 mM NaCl, 150 mM KCl, 2 mM $MgCl_2$). The concentration of RNA and oligomers were 1.0 µM each. The electrophoretic mobility assay was performed on 15% polyacrylamide gel at 9.4V/cm for 1 h at room temperature.

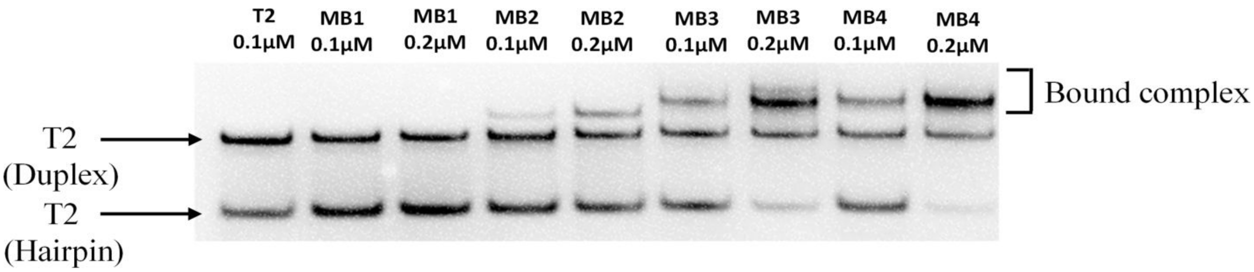

Figure S26. Results of EMSA binding studies with T2. The samples were prepared by mixing pre-annealed RNA and the indicated oligomers and incubated at 37 °C for 1 h in a physiologically relevant buffer (10 mM NaPi, 137 mM NaCl, 150 mM KCl, 2 mM $MgCl_2$). The concentration of RNA and oligomers were 1.0 μM each. The electrophoretic mobility assay was performed on 15% polyacrylamide gel at 9.4V/cm for 1 h at room temperature.

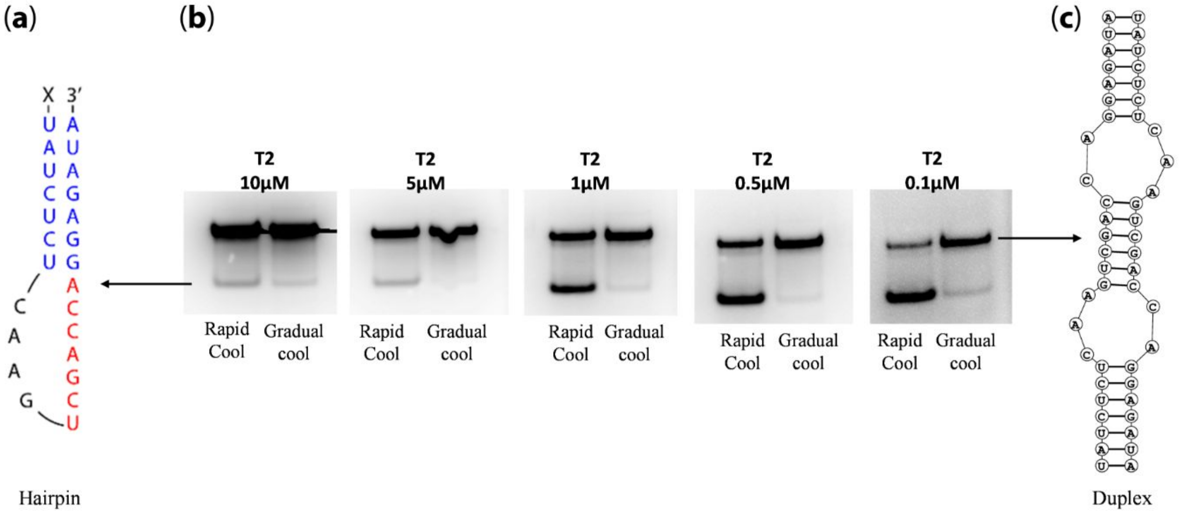

Figure S27. Analysis of the conformation of T2. (**a**) Structure of the intramolecular stem-loop, (**b**) EMSA results of the T2 samples prepared under different conditions, and (**c**) structure of the intermolecular duplex. The samples were prepared in a physiologically relevant buffer and annealed 95° C for 5 min, followed by two cooling methods—rapid quenching and gradual cooling.

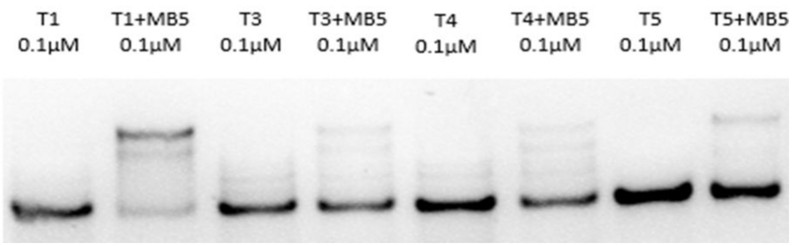

Figure S28. The effects of single-base RNA mismatches on MB5 binding. The samples were prepared by mixing pre-annealed RNA and the indicated oligomers and incubated at 37 °C for 1 h in a physiologically relevant buffer (10 mM NaPi, 137 mM NaCl, 150 mM KCl, 2 mM $MgCl_2$). The concentration of RNA and oligomers were 1.0 μM each. The electrophoretic mobility assay was performed on 15% polyacrylamide gel at 9.4V/cm for 1 h at room temperature.

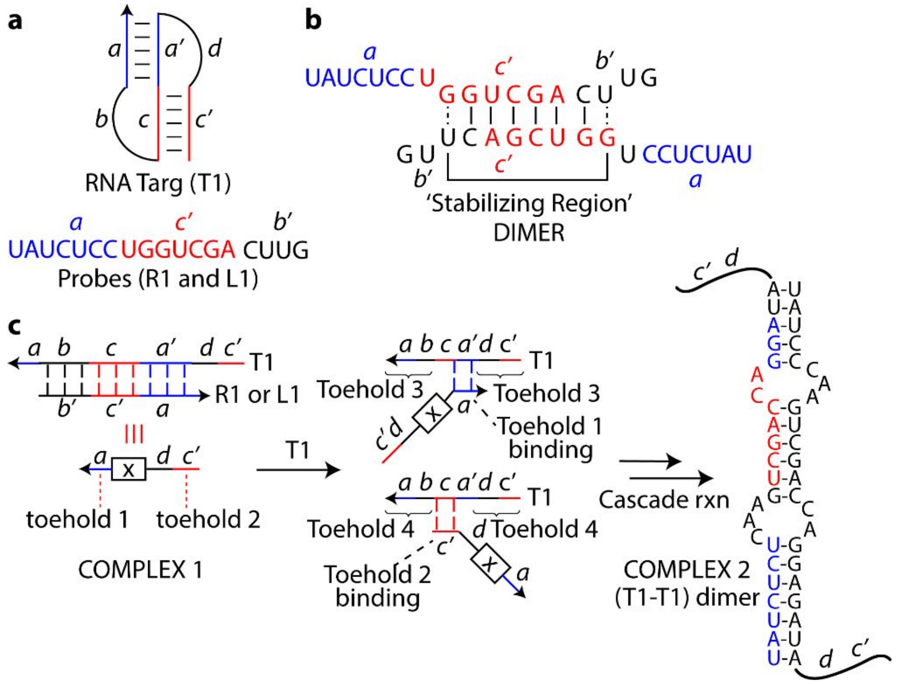

Figure S29. Probe hybridization pathways. (**a**) Schematic depiction of the RNA target and the probe sequence. (**b**) Formation of probe dimers. (**c**) A cascade hybridization chain reaction catalyzed by probe binding to RNA target, ultimately leading to the formation of a stable T1-T1 dimer.

(a)

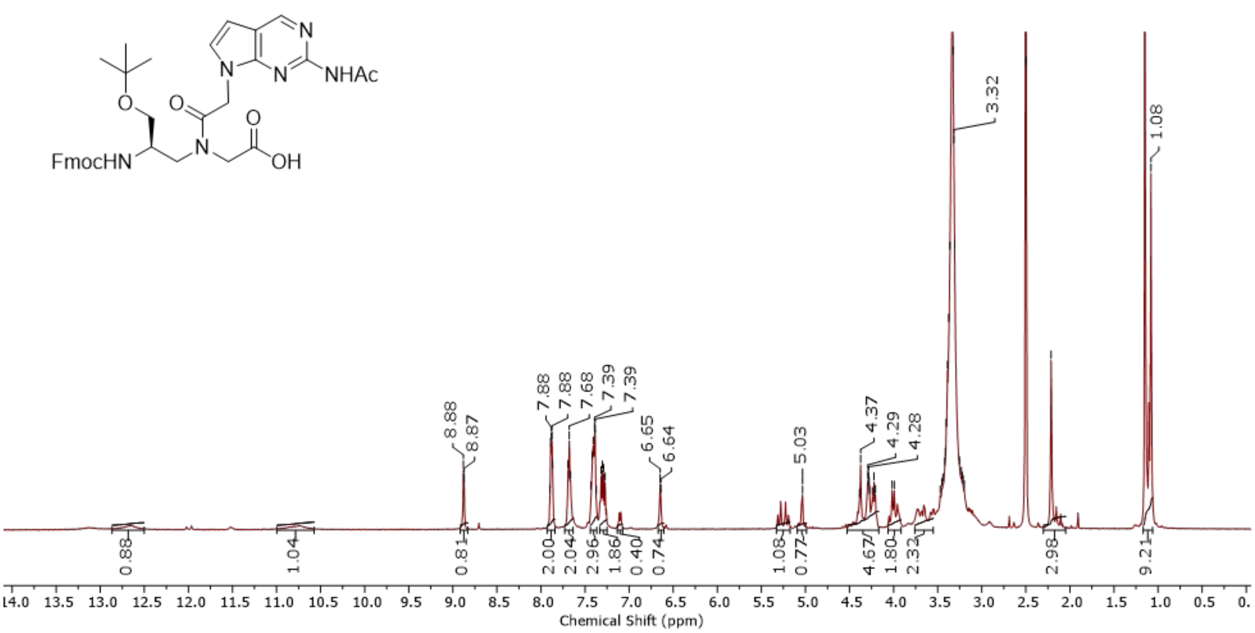

NHAc
FmocHN
OH
Chemical Shift (ppm)

(b)

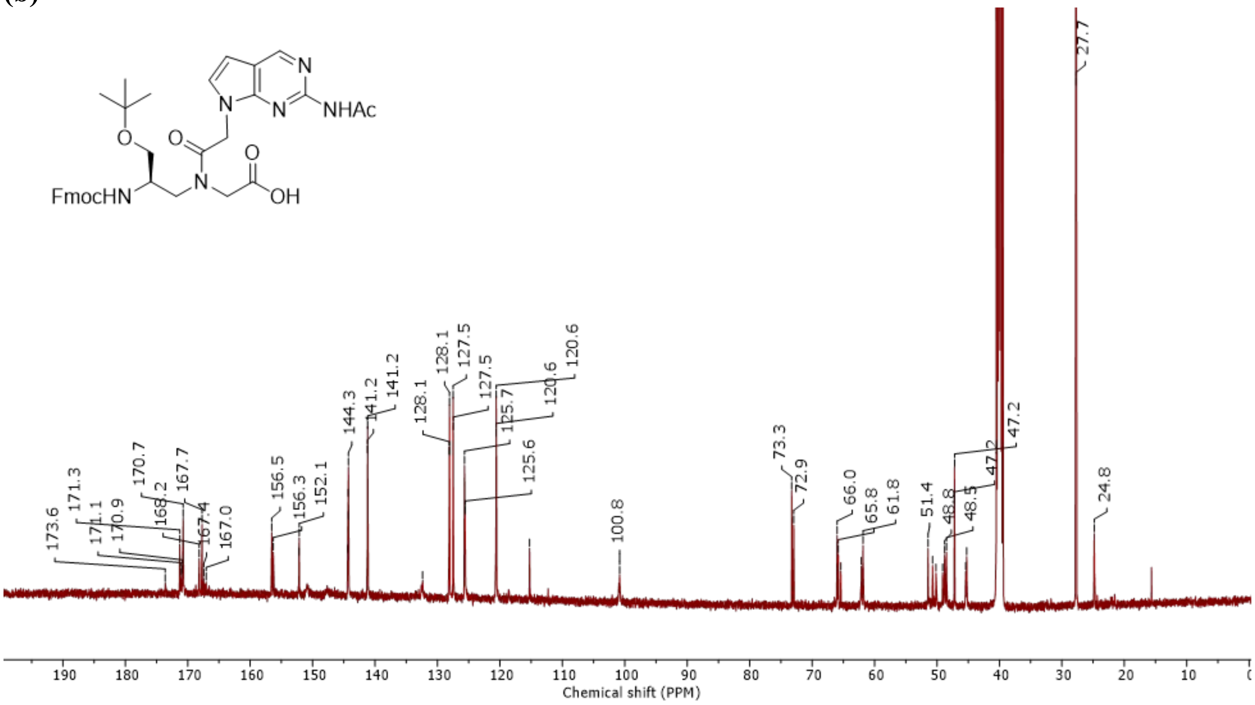

NHAc
FmocHN
OH
Chemical shift (PPM)

(c)

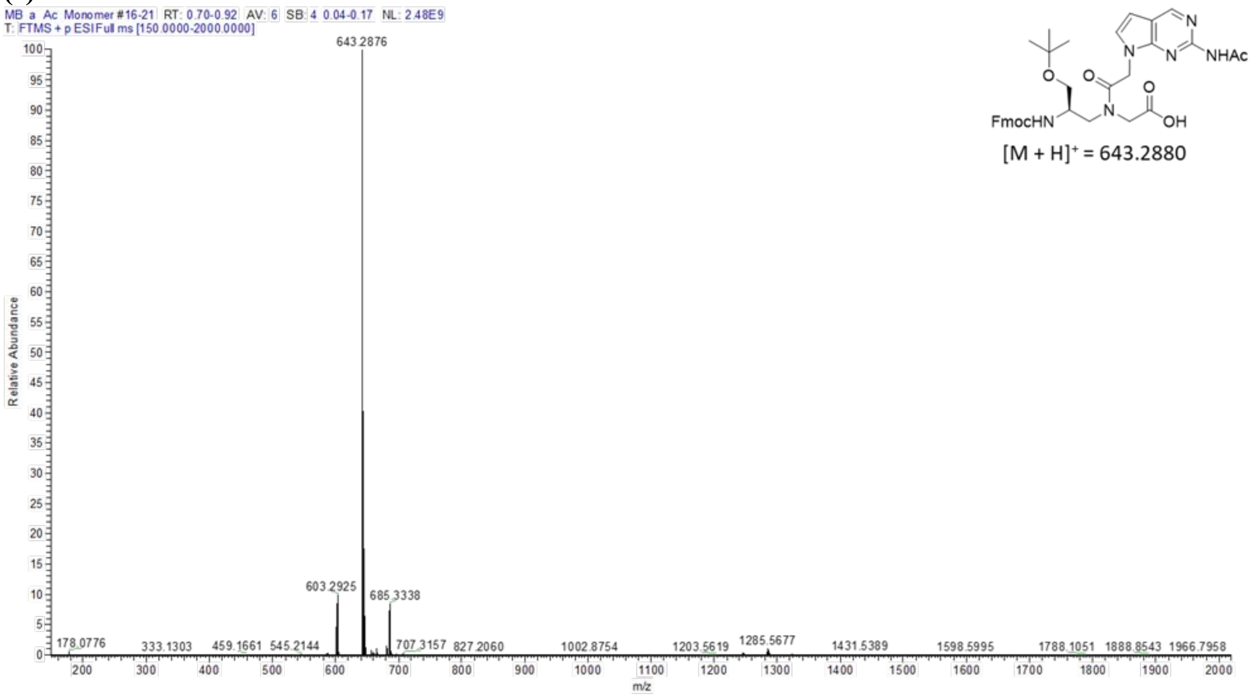

Figure S30. Spectroscopic data for compound **1a (Modified a(Ac) Monomer)**: (**a**) ^{1}H NMR (500.13 MHz, DMSO-d$_6$), and (**b**) ^{13}C NMR (125.74 MHz, DMSO-d$_6$), and (**c**) HRMS (ESI-MS).

(a)

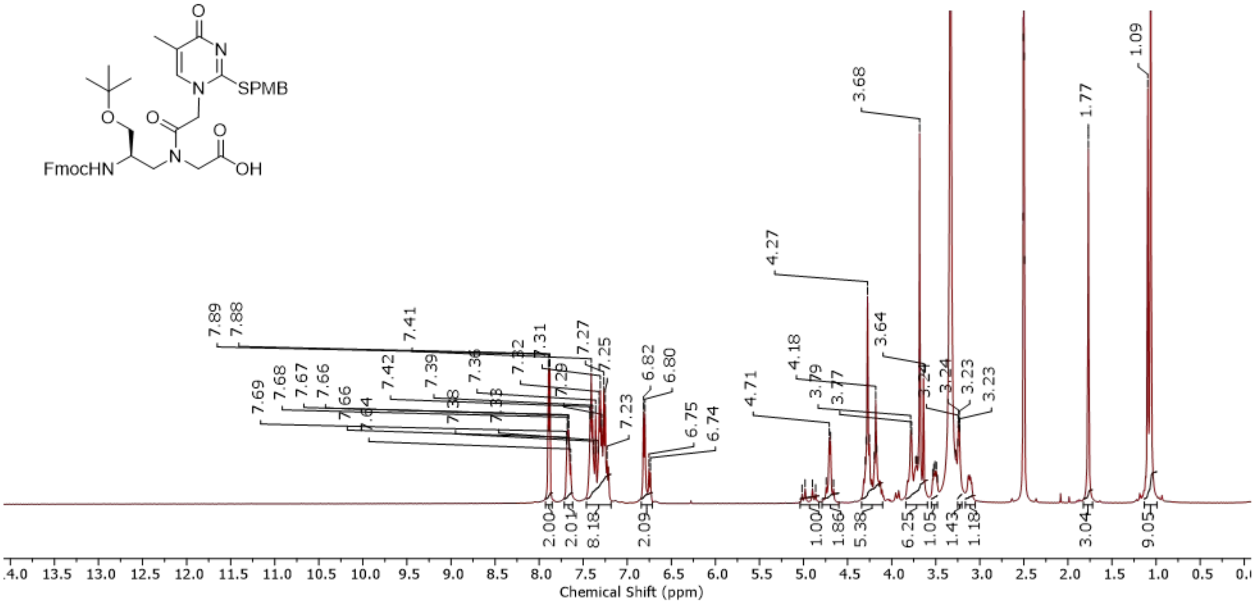

O
N
SPMB
N
O
O
FmocHN
N
O
OH
3.68
1.77
1.09
4.27
7.89
7.88
7.41
7.31
7.27
7.25
6.82
6.80
3.64
4.18
3.79
3.77
3.24
3.23
3.23
7.69
7.68
7.67
7.66
7.42
7.39
7.23
6.75
6.74
4.71
2.00
2.01
8.18
2.09
1.00
1.86
5.38
6.25
1.05
1.43
1.18
3.04
9.05
Chemical Shift (ppm)

(b)

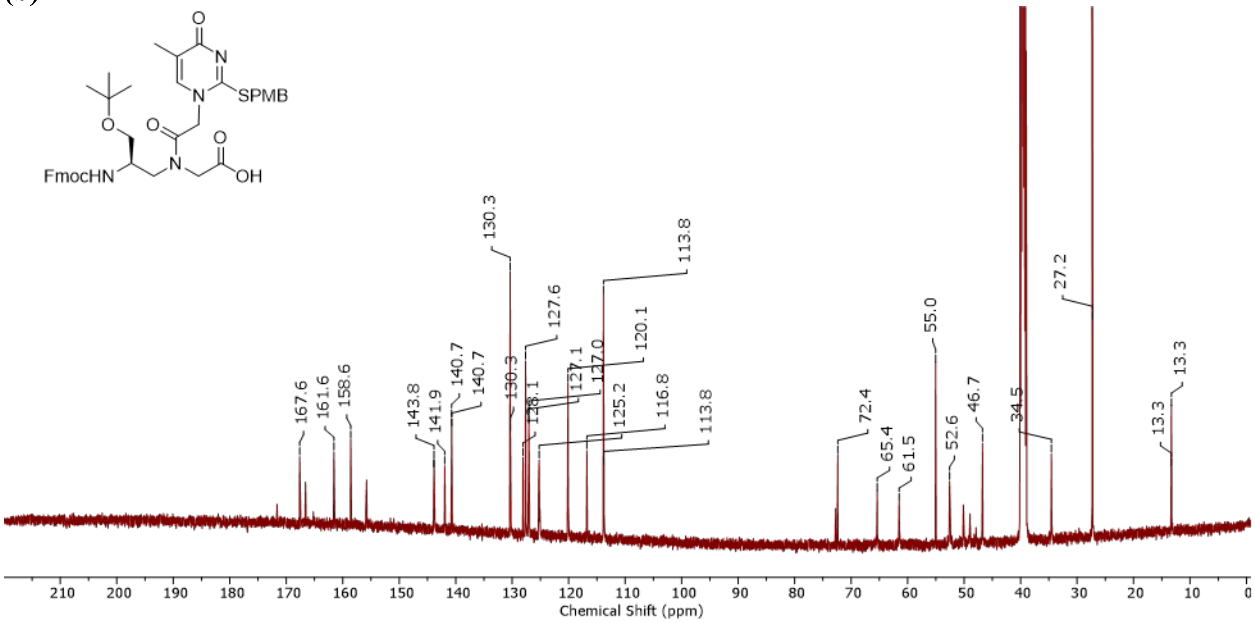

O
N
SPMB
N
O
O
FmocHN
N
O
OH
130.3
113.8
127.6
27.2
55.0
120.1
140.7
140.7
130.3
127.0
13.3
167.6
161.6
158.6
143.8
141.9
125.2
116.8
113.8
72.4
65.4
61.5
52.6
46.7
34.5
13.3
Chemical Shift (ppm)

(c)

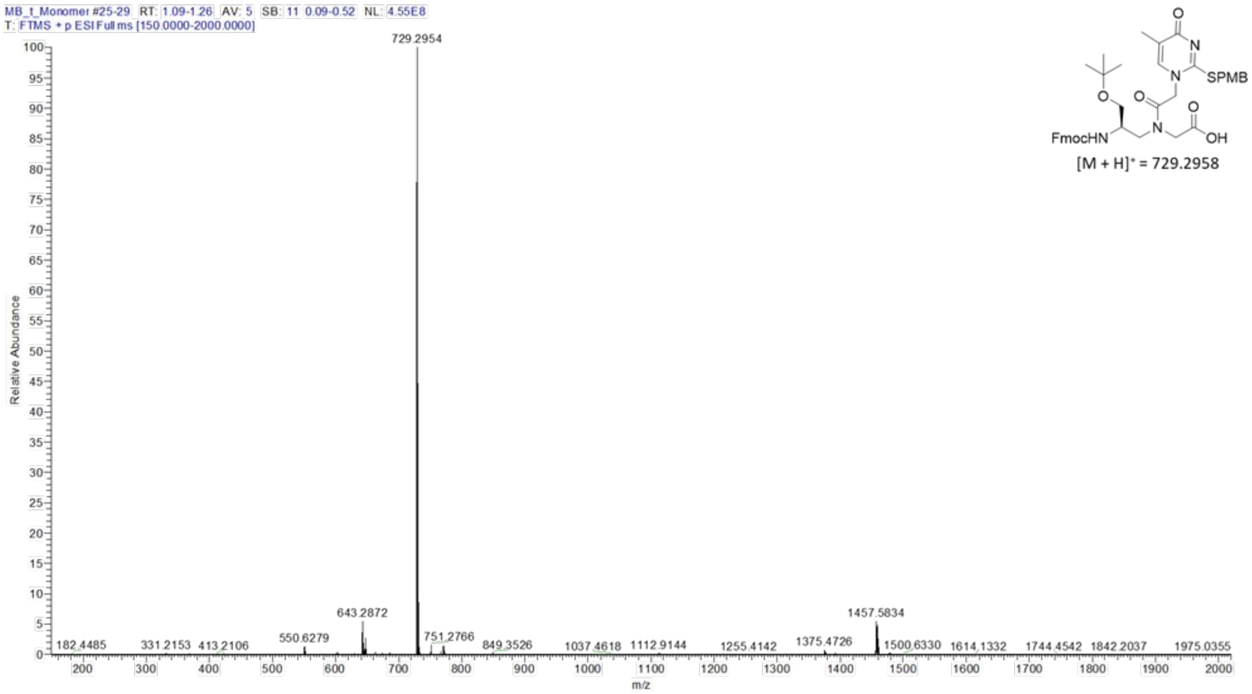

Figure S31. Spectroscopic data for compound **1b (Modified t(PMB) Monomer)**: (**a**) ^{1}H NMR (500.13 MHz, DMSO-d_6), and (**b**) ^{13}C NMR (125.74 MHz, DMSO-d_6), and (**c**) HRMS (ESI-MS).

(a)

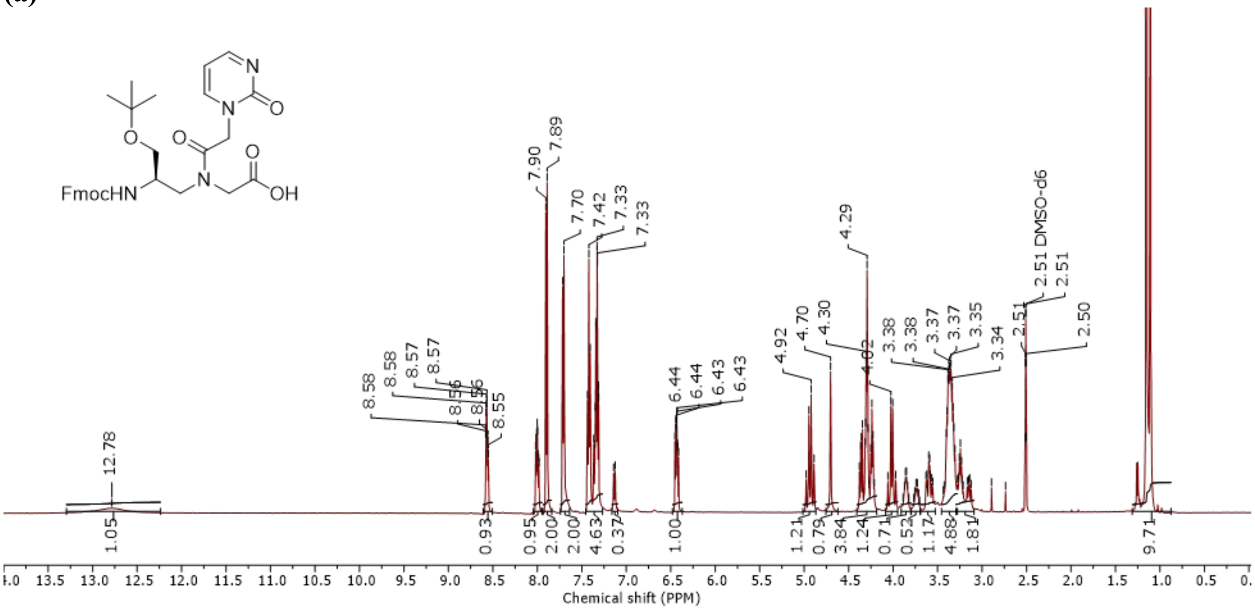
FmocHN
OH
Chemical shift (PPM)
2.51 DMSO-d6

(b)

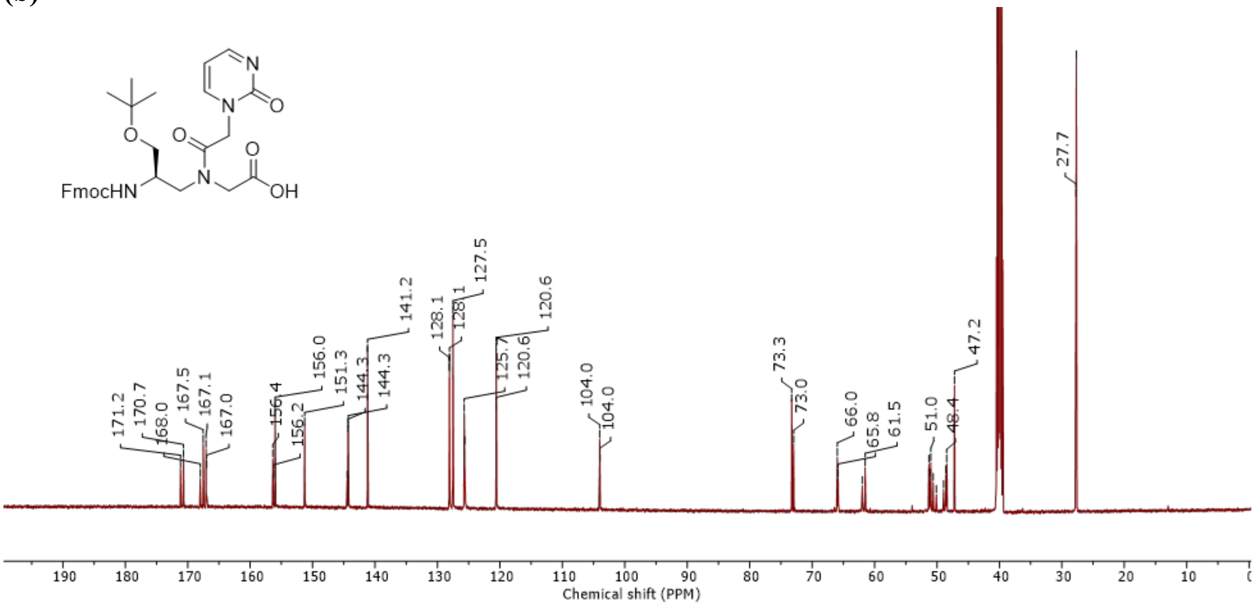
FmocHN
OH
Chemical shift (PPM)

(c)

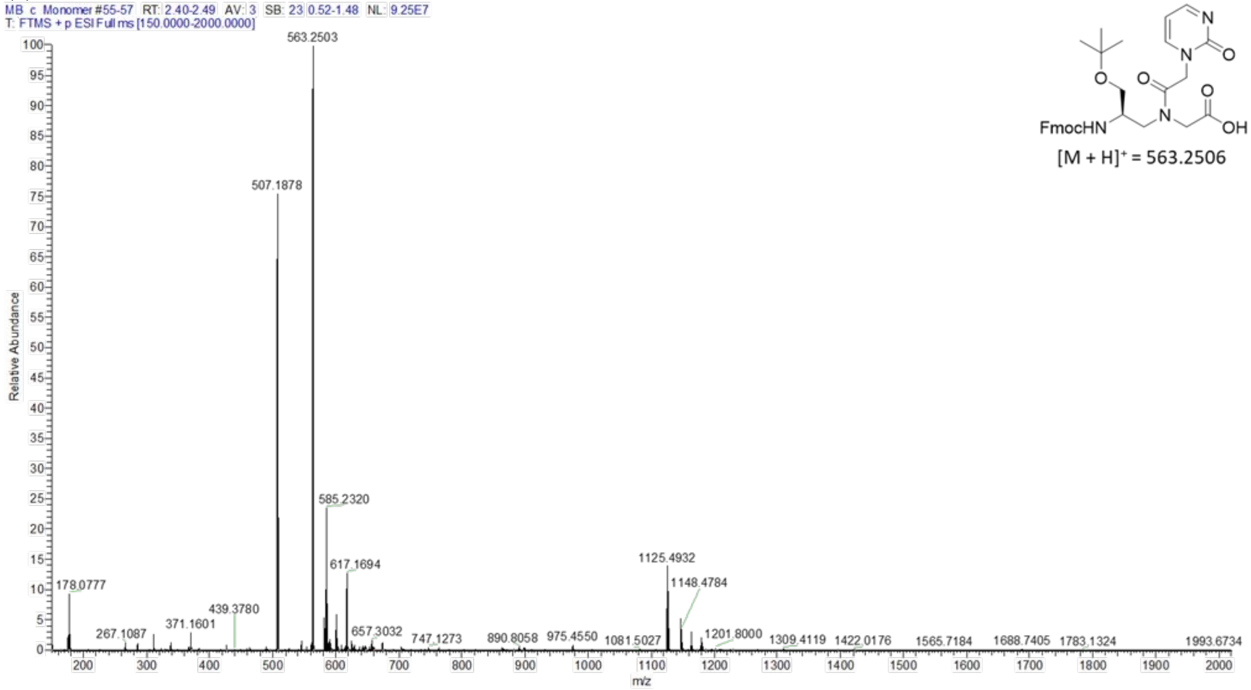

Figure S32. Spectroscopic data for compound **1c (Modified c Monomer)**: (**a**) ^{1}H NMR (500.13 MHz, DMSO-d_6), and (**b**) ^{13}C NMR (125.74 MHz, DMSO-d_6), and (**c**) HRMS (ESI-MS).

(a)

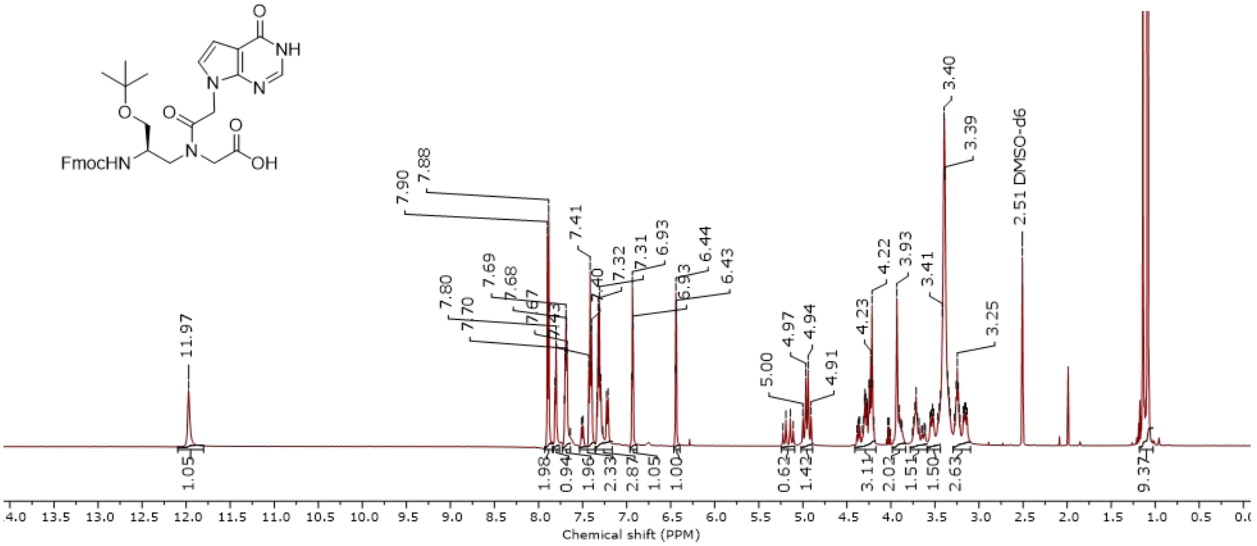
FmocHN
OH
NH
Chemical shift (PPM)
2.51 DMSO-d6
11.97
7.90
7.88
7.80
7.70
7.69
7.68
7.67
7.43
7.41
7.40
7.32
7.31
6.93
6.93
6.44
6.43
5.00
4.97
4.94
4.91
4.23
4.22
3.93
3.41
3.40
3.39
3.25

(b)

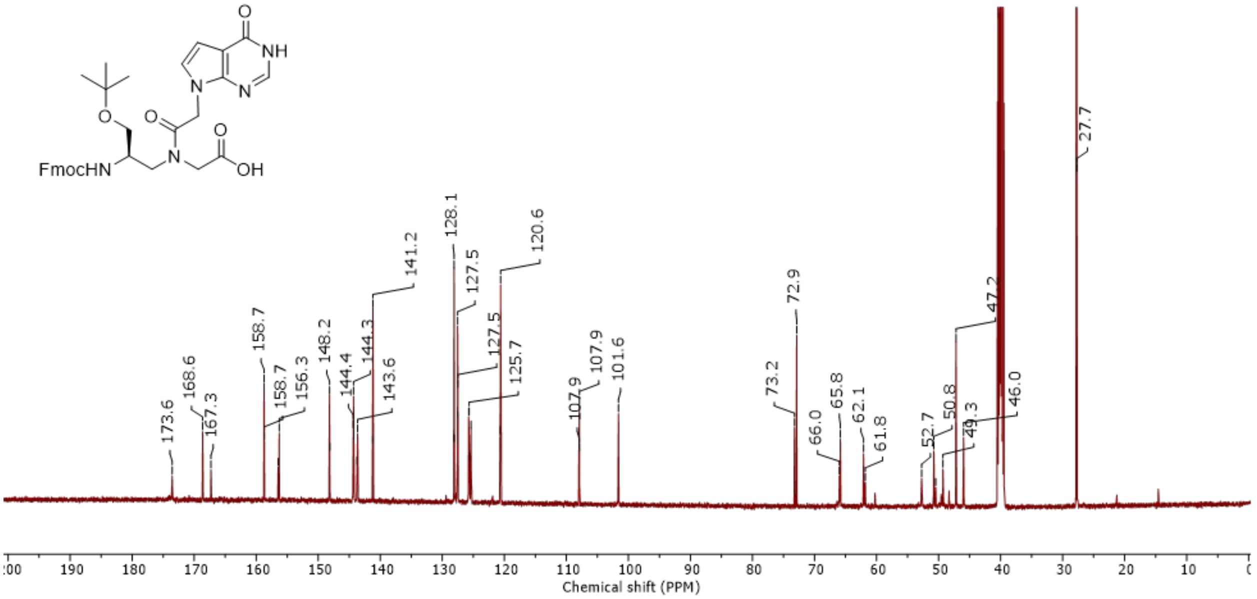
FmocHN
OH
NH
Chemical shift (PPM)
173.6
168.6
167.3
158.7
158.7
156.3
148.2
144.4
144.3
143.6
141.2
128.1
127.5
127.5
125.7
120.6
107.9
107.9
101.6
73.2
72.9
66.0
65.8
62.1
61.8
52.7
50.8
49.3
47.2
46.0
27.7

(c)

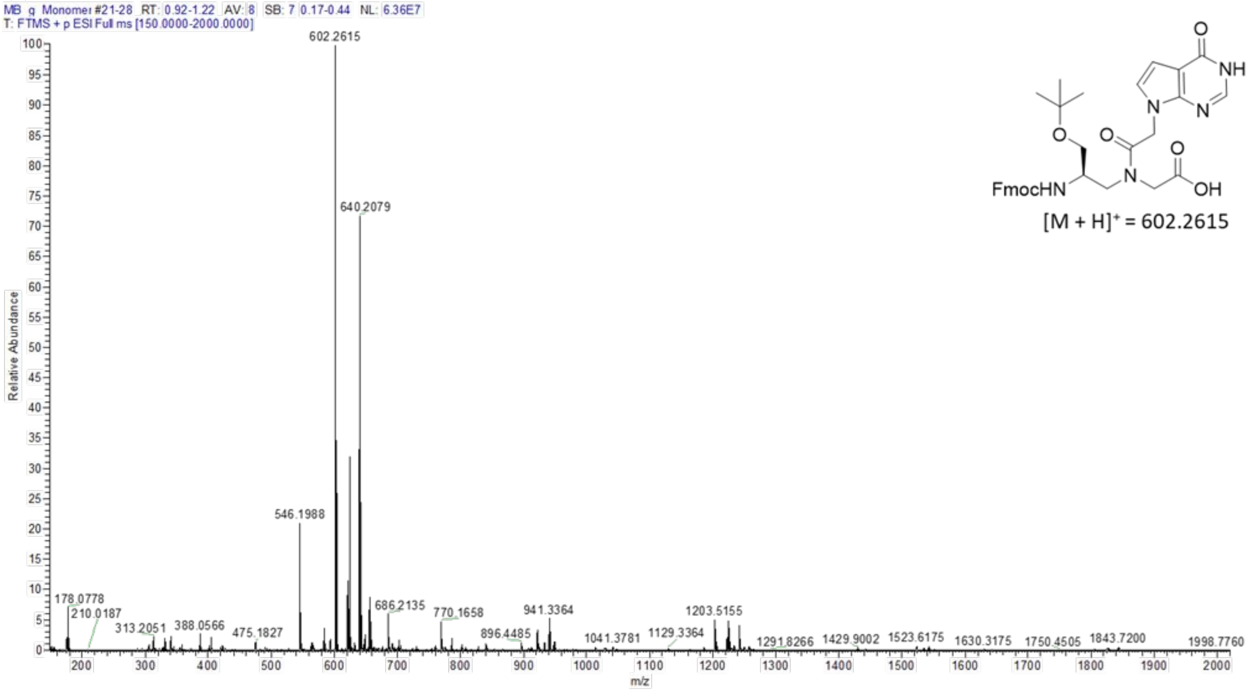

Figure S33. Spectroscopic data for compound **1d (Modified g Monomer)**: (**a**) ^{1}H NMR (500.13 MHz, DMSO-d_6), and (**b**) ^{13}C NMR (125.74 MHz, DMSO-d_6), and (**c**) HRMS (ESI-MS).

(a)

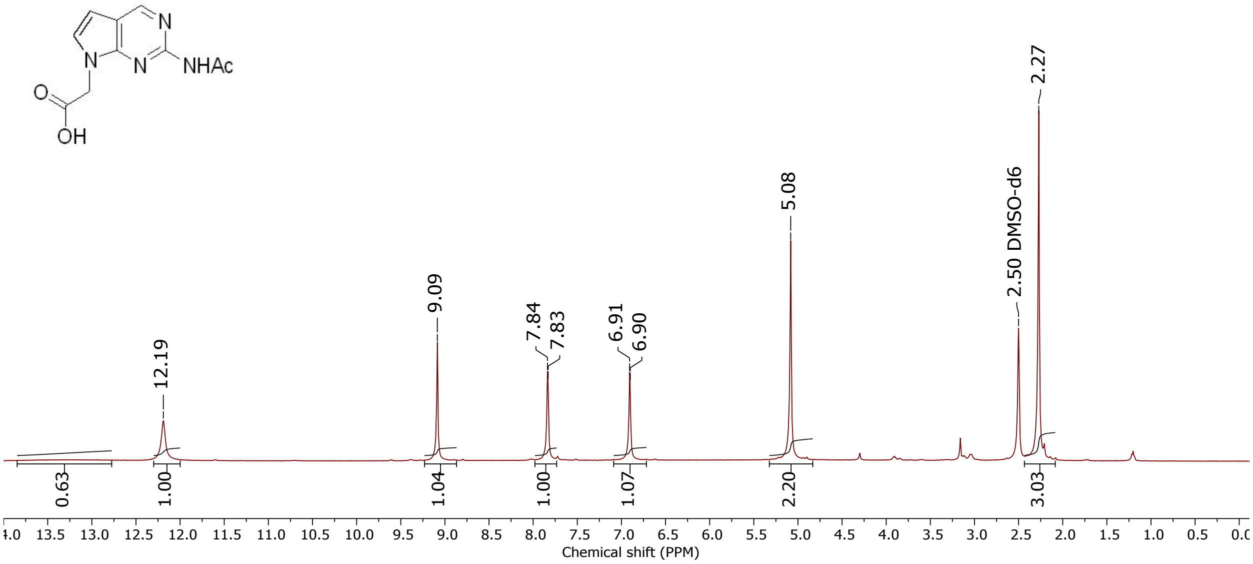
N
N
N
NHAc
O
OH
12.19
9.09
7.84
7.83
6.91
6.90
5.08
2.50 DMSO-d6
2.27
0.63
1.00
1.04
1.00
1.07
2.20
3.03
Chemical shift (PPM)

(b)

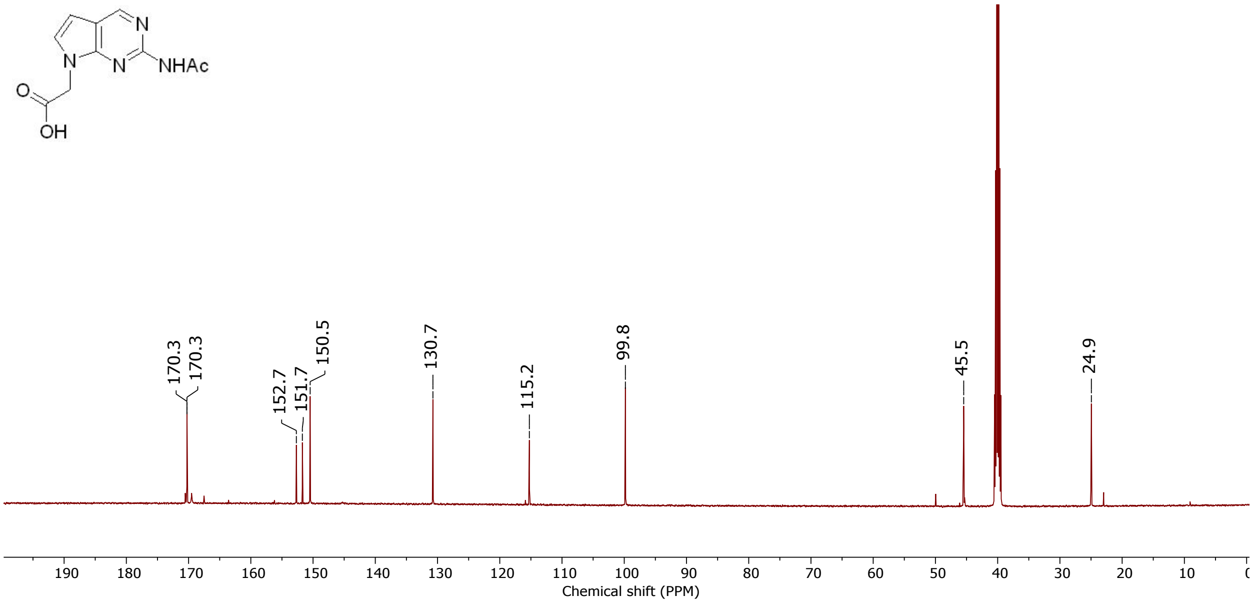
N
N
N
NHAc
O
OH
170.3
170.3
152.7
151.7
150.5
130.7
115.2
99.8
45.5
24.9
Chemical shift (PPM)

(c)

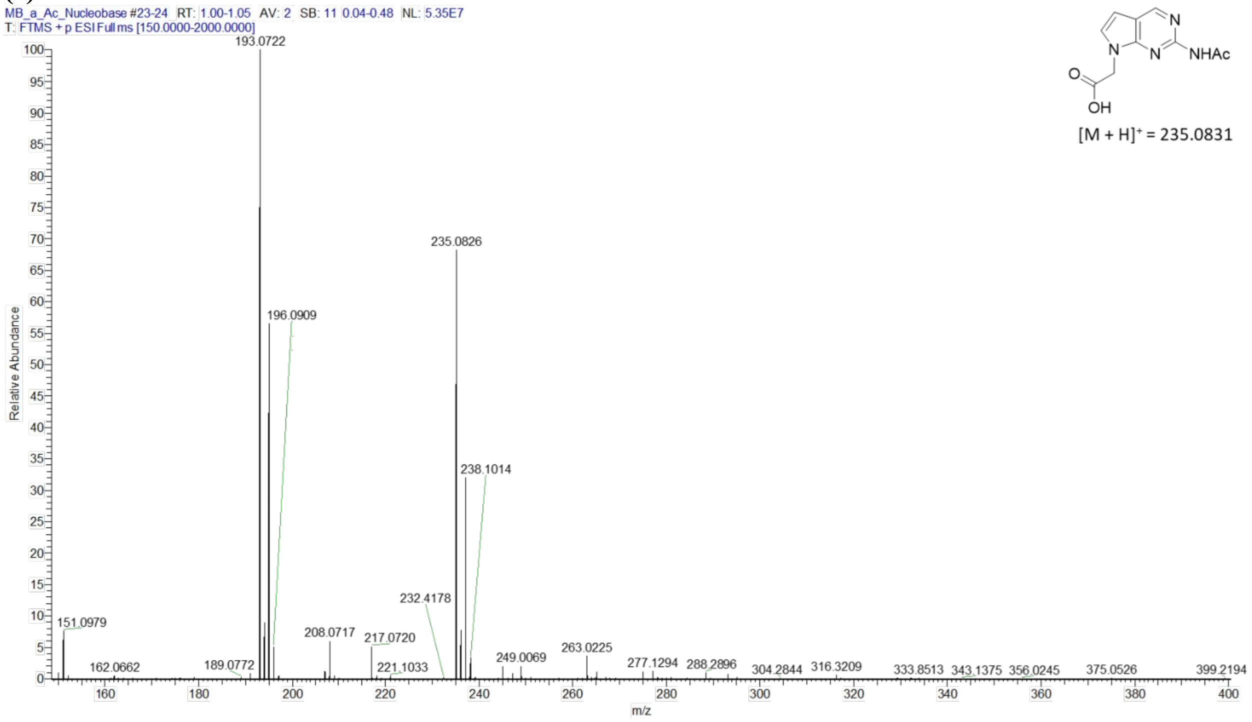

Figure S34. Spectroscopic data for **Modified a$^{(Ac)}$ Nucleobase**: (**a**) ^{1}H NMR (500.13 MHz, DMSO-d_6), and (**b**) ^{13}C NMR (125.74 MHz, DMSO-d_6), and (**c**) HRMS (ESI-MS).

(a)

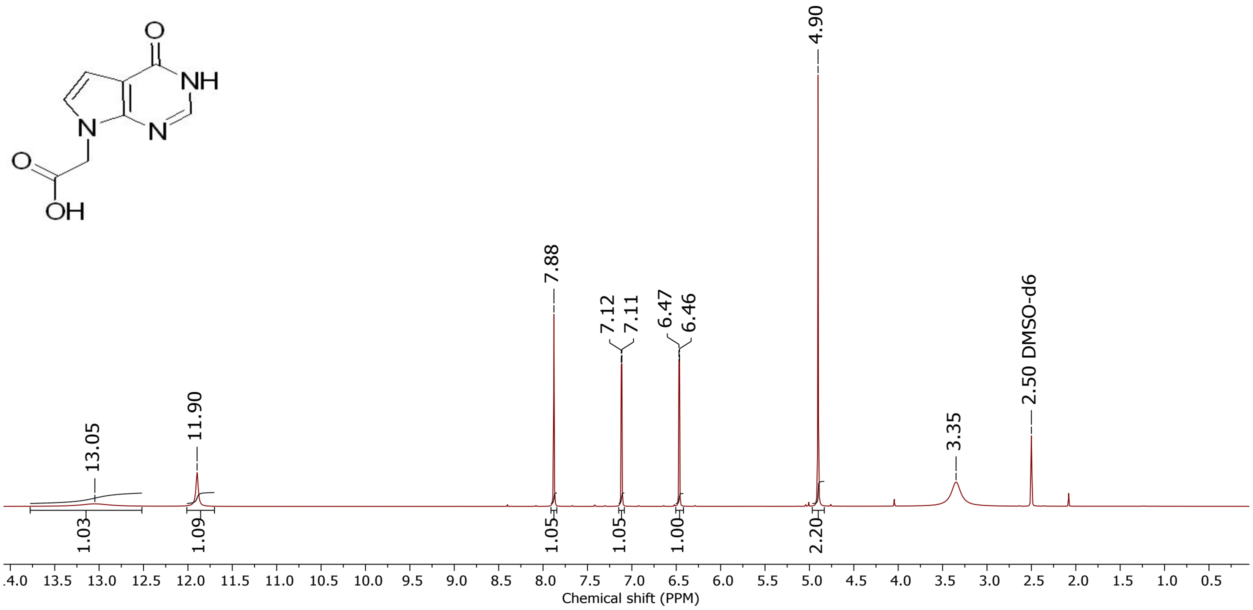

O
NH
N
N
O
OH
4.90
7.88
7.12
7.11
6.47
6.46
2.50 DMSO-d6
11.90
13.05
3.35
1.03
1.09
1.05
1.05
1.00
2.20
Chemical shift (PPM)

(b)

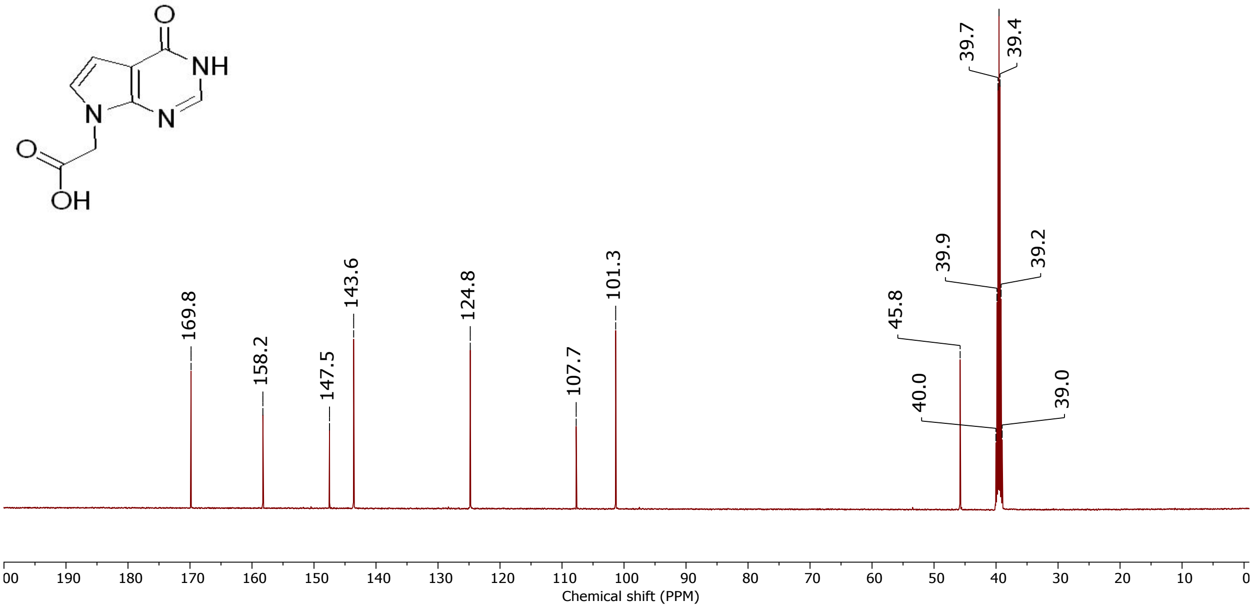

O
NH
N
N
O
OH
39.7
39.4
39.9
39.2
169.8
158.2
147.5
143.6
124.8
107.7
101.3
45.8
40.0
39.0
Chemical shift (PPM)

(c)

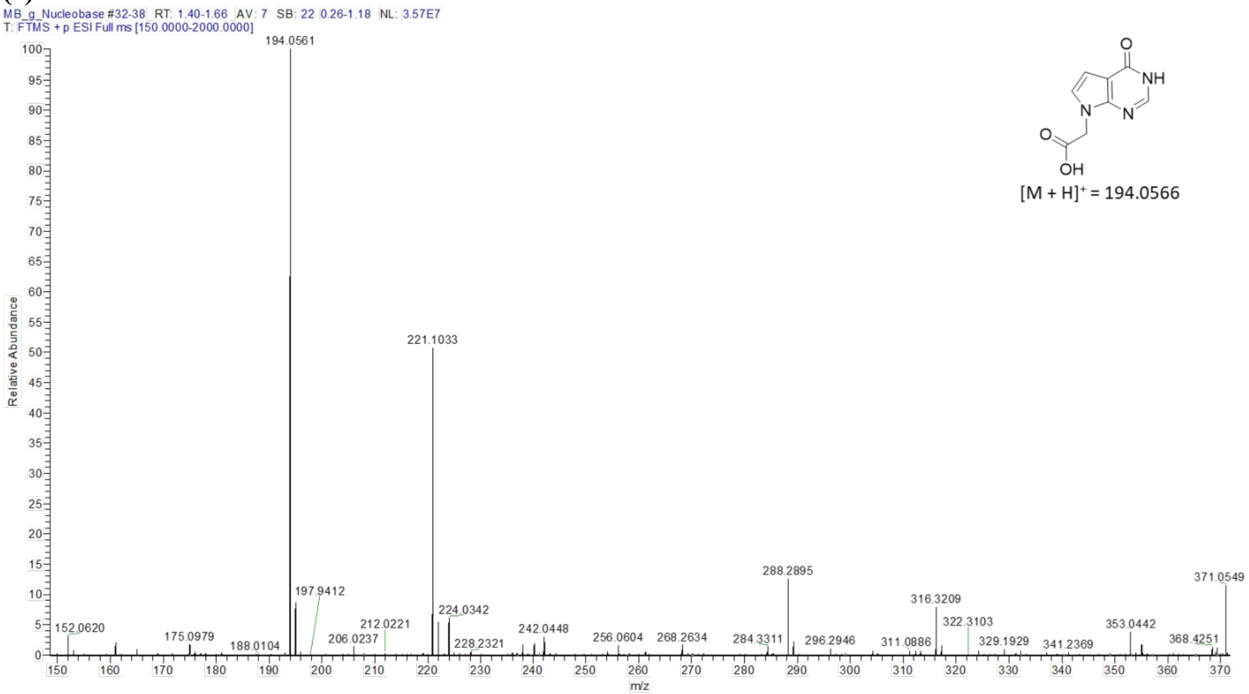

Figure S35. Spectroscopic data for **Modified g Nucleobase**: (**a**) ^{1}H NMR (500.13 MHz, DMSO-d$_6$), and (**b**) ^{13}C NMR (125.74 MHz, DMSO-d$_6$), and (**c**) HRMS (ESI-MS).

EXPERIMENTAL PROCEDURES

UV-melting Analysis

The samples were prepared in a stoichiometric ratio at 2.5 μM strand concentration each in 1x PBS buffer by mixing ligands with RNA targets, annealed by incubation at 90 °C for 5 min followed by a gradual cooling to room temperature. UV melting curves were collected using Agilent Cary UV-Vis 300 spectrometer equipped with a thermoelectrically controlled multi-cell holder. UV-melting spectra were collected by monitoring UV-absorption at 260 nm from 25 to 95 °C in the heating runs, and from 95 to 25 °C in the cooling runs, both at the rate of 1 °C per min. The recorded spectra were smoothed using a 20-point adjacent averaging algorithm. The first derivatives of the melting curves were taken to determine the melting temperatures of the complex.

EMSA Analysis

All samples were prepared by incubating the pre-annealed RNAs with respective probes in physiologically relevant buffer (10 mM NaPi, 137 mM NaCl, 150 mM KCl, 2 mM $MgCl_2$; pH 7.4), at 37 °C temperature. RNAs were annealed by heating to 90 °C for 5 min, followed by rapid cooling to 0 °C, and then allowing to reach room temperature. The samples were then loaded onto 15% Polyacrylamide-gel (PAGE) with 1X Tris-borate buffer and electrophoretically separated. The gels were visualized by UV-Transilluminator, and the relative amounts of the bound complexes were quantified by ImageJ.

Oligomer Synthesis

Fmoc solid-phase synthesis was employed to prepare all oligomers using a PurePep Chorus peptide synthesizer, incorporating several specific modifications. In this process, the N-terminal amine was capped by treating the resin with a 5% capping solution (2 mL for 100 mg resin) at room temperature, a step repeated two times with each cycle lasting two minutes. Deprotection of the Fmoc group was subsequently carried out using 20% piperidine, and the final cleavage of the oligomers was achieved with a mixture consisting of 95% TFA and 5% m-cresol.

Chemical Synthesis of Monomers

General protocol for Coupling: To a mixture of a nucleobase (1.00 eq. 10.00 mmol) and DIEA (2.00 eq, 20.00 mmol) in anhydrous DMF (50 mL, 0.2 M) was added HBTU (1.00 eq., 10.00 mmol) in one portion at room temperature. After 5 min at room temperature, Fmoc-^{L}Ser(^{t}Bu)-γPNA methyl ester HCl salt (1.00 eq.,10.00 mmol), obtained from Dr. NS Lab, was added to the above reaction mixture. TLC monitored the progress of the reaction. After consuming the starting material (Note: Sometimes heating requires up to 40 °C in an oil-bath for consumption of starting material), DMF was removed under vacuum at 45 °C in water bath. To the resultant crude material, saturated sodium bicarbonate (70 ml) and EA (100 ml) were added, and the organic layers (2x) were separated from the water layer using a separating funnel. The combined organic layer was dried, removed, and the acquired crude was purified by flash silica gel column chromatography. Yield: 64-73% for methyl ester monomers.

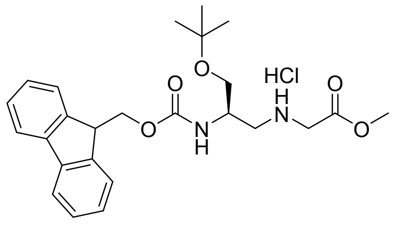

Fmoc-^{L}Ser(^{t}Bu)-γPNA methyl ester HCl salt

General protocol for monomers: To a stirred solution of $CaCl_2 \cdot 2H_2O$ (20.00 eq., 100.00 mmol) and the methyl ester monomer (1 eq., 5.00 mmol) was prepared in an IPA:Water mixture (7:3, 0.8 M). To this solution, 1 M NaOH (2 eq., 10.00 mmol) was added dropwise over 5 min. The reaction mixture was stirred at room temperature for an additional 3 hrs, with TLC confirming that (>85%), of the starting material had been consumed. The mixture was then acidified to pH 4–5 using glacial acetic acid. After removing the solvent under vacuum, the crude product was redissolved in MeOH. The subsequent addition of water produced a white precipitate, which was collected by vacuum filtration to yield the pure monomer. Finally, the monomer was further purified by flash silica gel column chromatography using DCM:MeOH as the eluents as eluents to obtain pure monomer (>95% purity). Yield: 62-71% monomer.

Modified a$^{(Ac)}$ Nucleobase: To a DMF (dry, 741 mL, 0.2M) solution of 4-chloro-7H-pyrrolo[2,3-d]pyrimidin-2-amine (25.00 g, 148.29 mmol) was added cesium carbonate (4.80 g, 14.83 mmol) followed by potassium carbonate (20.49 g, 148.29 mmol) at room temperature under inert atmosphere. After 5 min, benzyl bromoacetate (18.84 mL, 163.12mmol) was added dropwise and the mixture was left for stirring for 20 hrs. After the completion of the reaction, solvent was removed under reduced pressure and 1N HCl were added. The formed precipitate was collected by vacuum filtration and further recrystallized from methanol to obtain the pure product: 30.00 g, 79.4%. To a solution of Benzyl 2-(2-amino-4-chloro-7H-pyrrolo [2,3-d]pyrimidin-7-yl)acetate (14.00 g, 39.02 mmol), triethyl amine (93.01 mL, 667.26 mL) and formic acid (13.32 mL, 353.14 mmol) in acetone (269 mL, 0.15M), was added palladium/charcoal (2.66 g, 2.50 mmol) at room temperature. The mixture was refluxed in an oil-bath for 4hr and further same equivalents of triethyl amine, formic acid and palladium/charcoal were added. The reaction mixture was cooled down after stirring overnight at 70 °C in an oil-bath and a small amount of water was added. The reaction mixture was filtered through a celite pad and washed with acetone under continuous flow of nitrogen. The acetone layer was further removed under vacuum to afford the final desired product as a white solid. Yield: 5.50 g, 60%. ^{1}H NMR (500.00 MHz, DMSO-d_6): δ 2.27 (s, 3H), 5.08 (s, 2H), 6.90 (d, J = 3.8 Hz, 1H), 7.83 (d, J = 3.8 Hz, 1H), 9.09 (s, 1H), 12.19 (s, 1H), 13.31 (s, 1H). ^{13}C{^{1}H} NMR (125.74 MHz, DMSO-d_6): δ 24.9, 45.5, 99.8, 115.2, 130.7, 150.5, 151.7, 152.7, 170.3 (2C). HRMS (ESI-MS) m/z: $[M + H]^+$ Calcd for $C_{10}H_{11}N_4O_3$ 235.0831; Found: 235.0826.

Modified g Nucleobase: To an ice-water bath solution of 4-chloro-7H-pyrrolo[2,3-d]pyrimidine (5.0 g, 32.56 mmol) in DMF (dry, 65 mL, 0.5M) was added sodium hydride (60%, 1.30 g, 32.56 mmol). After stirring at room temperature for 5 minutes, 1,1-dimethylethyl bromoacetate (4.81 mL, 32.56 mmol) was then added. The mixture was stirred at room temperature for 2 hours. Ethyl acetate was then added (100 mL) and washed with water (2×100 mL). The collected organic layers were dried over magnesium sulfate and removed on rotary evaporator. The obtained residue purified by column chromatography using ethyl acetate/hexane gradient (5%-40% over 20 min) to obtain the title compound as a light-yellow solid. Yield of the product (8.00 g, 92%). A suspension of t-Butyl 2-(4-chloro-7H-pyrrolo [2,3-d]pyrimidin-7-yl)acetate (6.00 g, 22.40 mmol) in 0.5N sodium hydroxide (4.48 g, 112.06 mmol) solution (224 mL) was heated at reflux for 6 hr. After complete consumption of the starting material monitored by TLC, the reaction mixture was neutralized with 0.5N HCl and the resultant solution was kept in the refrigerator for overnight. The obtained colorless solid was collected by vacuum filtration and washed with water to afford pure product. Yield of the product: 3.50 g, 81% White solid. ^{1}H NMR (500.00 MHz, DMSO-d_6): δ 4.90 (s, 2H), 6.47 (d, J = 3.3 Hz, 1H), 7.12 (d, J = 3.4 Hz, 1H), 7.88 (s, 1H), 11.90 (s, 1H), 13.05 (s, 1H). ^{13}C{^{1}H} NMR (125.74 MHz, DMSO-d_6): δ 45.8, 101.3, 107.7, 124.8, 143.6, 147.5, 158.2, 169.8. HRMS (ESI-MS) m/z: $[M + H]^+$ Calcd for $C_8H_8N_3O_3$ 194.0566; Found: 194.0561.

Modified a(Ac) Monomer (1a):

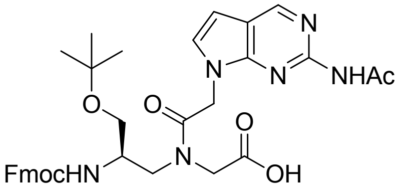

This compound was prepared using the above-mentioned procedure for methyl ester monomer a(Ac) (3.28 g, 5.00 mmol) as the starting materials. White solid. Yield: 1.54 g, 64%. ^{1}H NMR (500.00 MHz, DMSO-d_6): δ 1.11 (d, J = 34.1 Hz, 9H), 2.21 (s, 3H), 3.40 – 3.78 (m, 3H), 3.89 – 4.07 (m, 2H), 4.16 – 4.53 (m, 5H), 4.97 – 5.10 (m, 1H), 5.15 – 5.35 (m, 1H), 6.64 (d, J = 3.8 Hz, 1H), 7.04 – 7.49 (m, 6H), 7.68 (t, J = 7.3 Hz, 2H), 7.88 (dt, J = 4.1, 7.8 Hz, 2H), 8.88 (d, J = 4.8 Hz, 1H), 10.75 (s, 1H), 12.66 (s, 1H). ^{13}C NMR (125.74 MHz, DMSO-d_6): δ 24.8, 27.7, 27.7, 45.2, 45.4, 47.2, 47.2, 48.5, 48.8, 49.1, 50.2, 50.7, 51.4, 61.8, 62.1, 65.4, 65.8, 66.0, 72.9, 73.3, 100.8, 115.3, 120.6, 120.6, 125.6, 125.7, 127.5, 127.5, 128.1, 128.1, 132.4, 141.2, 141.2, 144.2, 144.3, 144.3, 144.4, 152.1, 152.2, 156.3, 156.5, 167.0, 167.4, 167.7, 168.2, 170.7, 170.9, 171.1, 171.3, 173.6. HRMS (ESI-MS) m/z: [M + H]$^{+}$ Calcd for $C_{34}H_{39}N_6O_7$ 643.2880; Found: 643.2876.

Modified t Monomer (1b):

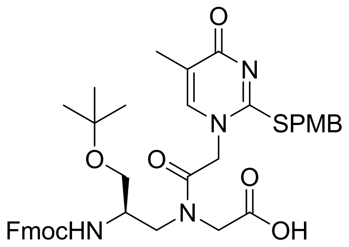

The compound (**1b**) was prepared using the above-mentioned procedure for methyl ester monomer t (11.14 g, 15.00 mmol) as the starting materials. White solid. Yield: 7.76 g, 71%. ^{1}H NMR (500.00 MHz, DMSO-d_6): δ 1.08 (d, J = 16.8 Hz, 9H), 1.77 (s, 3H), 3.03 – 3.27 (m, 3H), 3.51 (dd, J = 7.1, 13.9 Hz, 1H), 3.59 – 3.87 (m, 6H), 4.06 – 4.35 (m, 5H), 4.70 (d, J = 15.0 Hz, 2H), 4.94 (d, J = 20.0 Hz, 1H), 6.78 (dd, J = 8.2, 31.2 Hz, 2H), 7.33 (ddt, J = 8.6, 29.7, 50.9 Hz, 8H), 7.59 – 7.73 (m, 2H), 7.89 (d, J = 7.5 Hz, 2H). ^{13}C NMR (125.74 MHz, DMSO-d_6): δ 13.3, 13.3, 27.2, 27.3, 34.5, 46.7, 52.6, 55.0, 61.5, 65.4, 72.4, 113.8, 113.8, 116.8, 120.1, 125.2, 127.0, 127.1, 127.6, 128.1, 130.3, 130.3, 140.7, 140.7, 141.9, 143.8, 143.9, 158.6, 161.6, 167.6. HRMS (ESI-MS) m/z: [M + H]$^{+}$ Calcd for $C_{39}H_{47}N_4O_8S$ 729.2958; Found: 729.2954.

Modified c Monomer (1c):

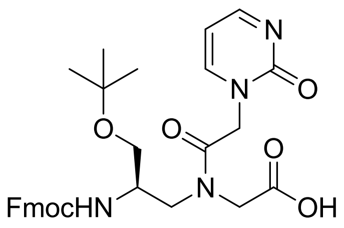

This compound was prepared using the above-mentioned procedure for methyl ester monomer c (5.77 g, 10.00 mmol) as the starting materials. White solid. Yield: 3.71 g, 66%. ^{1}H NMR (500.00 MHz, DMSO-d_6): δ 1.11/1.15 (s, 9H), 3.14 (dd, J = 8.2, 13.6 Hz, 1H), 3.19 – 3.29 (m, 1H), 3.28 – 3.44 (m, 2H), 3.59 (td, J = 5.5, 16.4, 17.3 Hz, 1H), 3.73 (td, J = 5.6, 13.3 Hz, 1H), 3.81 – 3.91 (m, 1H), 3.95 – 4.08 (m, 1H), 4.17 – 4.45 (m, 4H), 4.70 (s, 1H), 4.82 – 5.04 (m, 1H), 6.43 (ddd, J = 4.1, 6.5, 9.4 Hz, 1H), 7.10 – 7.52 (m, 5H), 7.71 (d, J = 7.5 Hz, 2H), 7.90 (d, J = 7.5 Hz, 2H), 8.00 (ddd, J = 2.9, 6.5, 10.7 Hz, 1H), 8.57 (ddd, J = 2.8, 4.2, 7.6 Hz, 1H), 12.78 (s, 1H). ^{13}C NMR (125.74 MHz, DMSO-d_6): δ 27.7, 27.7, 47.2, 48.4, 48.6, 48.9, 50.1, 50.6, 51.0, 51.1, 51.3, 61.5, 62.0, 65.8, 66.0, 73.0, 73.3, 104.0, 104.0, 120.6, 120.6, 125.6, 125.7, 127.5, 128.1, 128.1, 141.2, 144.3, 144.3, 144.5, 151.3, 156.0, 156.2, 156.4, 167.0, 167.1, 167.5, 168.0, 170.7, 171.2. HRMS (ESI-MS) m/z: $[M + H]^+$ Calcd for $C_{30}H_{35}N_4O_7$ 563.2506; Found: 563.2503.

Modified g Monomer (1d):

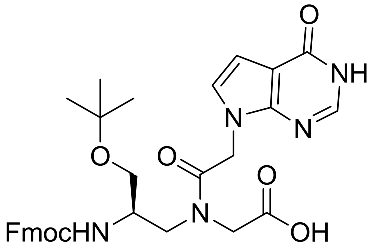

This compound was prepared using the above-mentioned procedure for methyl ester monomer g (6.15 g, 10.00 mmol) as the starting materials. White solid. Yield: 3.14 g, 65%. ^{1}H NMR (500.00 MHz, DMSO-d_6): δ 1.08/1.14 (s, 9H), 3.12 – 3.19 (m, 1H), 3.20 – 3.29 (m, 2H), 3.54 (dd, J = 6.2, 13.6 Hz, 1H), 3.59 – 3.77 (m, 2H), 3.92 (d, J = 14.0 Hz, 2H), 4.16 – 4.38 (m, 3H), 4.87 – 5.30 (m, 2H), 6.44 (t, J = 2.8 Hz, 1H), 6.93 (t, J = 3.5 Hz, 1H), 7.17 – 7.37 (m, 3H), 7.37 – 7.55 (m, 2H), 7.69 (dt, J = 4.0, 7.6 Hz, 2H), 7.81 (d, J = 6.0 Hz, 1H), 7.89 (d, J = 7.5 Hz, 2H), 11.97 (s, 1H). ^{13}C NMR (125.74 MHz, DMSO-d_6): δ 27.7, 27.8, 46.0, 47.2, 49.3, 50.5, 50.8, 52.7, 61.8, 62.1, 65.8, 66.0, 72.9, 73.2, 101.6, 107.9, 107.9, 120.6, 120.6, 125.4, 125.6, 125.7, 125.7, 127.5, 127.5, 127.6, 128.1, 128.1, 141.2, 141.2, 143.6, 143.7, 144.3, 144.3, 144.4, 148.1, 148.2, 156.3, 156.5, 158.7, 158.7, 167.3, 168.6, 173.6. HRMS (ESI-MS) m/z: $[M + H]^+$ Calcd for $C_{32}H_{34}N_5O_7$ 602.2615; Found: 602.2615.
